# New *Drosophila* models to uncover the intrinsic and extrinsic factors that mediate the toxicity of the human prion protein

**DOI:** 10.1242/dmm.049184

**Published:** 2022-05-04

**Authors:** Ryan R. Myers, Jonatan Sanchez-Garcia, Daniel C. Leving, Richard G. Melvin, Pedro Fernandez-Funez

**Affiliations:** 1 Department of Biomedical Sciences, University of Minnesota Medical School, Duluth Campus, Duluth, MN 55812, USA; 2 Departent of Neurology, University of Florida, Gainesville, FL 32611, USA

**Keywords:** Prion diseases, Prion protein, *Drosophila*, Transgenic models, Protective amino acids, Heat shock proteins, Unfolded protein response, PERK, ATF4 suppressors

## Abstract

Misfolding of the prion protein (PrP) is responsible for devastating neurological disorders in humans and other mammals. An unresolved problem in the field is unraveling the mechanisms governing PrP conformational dynamics, misfolding, and the cellular mechanism leading to neurodegeneration. The variable susceptibility of mammals to prion diseases is a natural resource that can be exploited to understand the conformational dynamics of PrP. Here we present a new fly model expressing human PrP with new, robust phenotypes in brain neurons and the eye. By using comparable attP2 insertions, we demonstrated the heightened toxicity of human PrP compared to rodent PrP along with a specific interaction with the amyloid-β peptide. By using this new model, we started to uncover the intrinsic (sequence/structure) and extrinsic (interactions) factors regulating PrP toxicity. We described PERK (officially known as EIF2AK3 in humans) and activating transcription factor 4 (ATF4) as key in the cellular mechanism mediating the toxicity of human PrP and uncover a key new protective activity for 4E-BP (officially known as Thor in *Drosophila* and EIF4EBP2 in humans), an ATF4 transcriptional target. Lastly, mutations in human PrP (N159D, D167S, N174S) showed partial protective activity, revealing its high propensity to misfold into toxic conformations.

## Abstract (precis)

**Summary:** New *Drosophila* models of prion diseases show the high toxicity of human PrP compared to rodent PrP and identify genetic modifiers and protective residues.

## INTRODUCTION

Prion diseases encompass a clinically heterogeneous class of brain disorders in humans, with direct molecular and pathological correlates in several other mammalian species (Mathiason, 2017; Zlotnik and Rennie, 1965). The main pathological features shared by prion diseases are spongiform degeneration of the brain and accumulation of insoluble prion protein (PrP; encoded by *PRNP*) (Colby and Prusiner, 2011; Scheckel and Aguzzi, 2018). PrP is a glycoprotein anchored to the extracellular aspect of the membrane not essential for survival (Sigurdson et al., 2019; Steele et al., 2007; Bueler et al., 1992). Other than humans, only ruminants suffer endemic prion diseases. Several mammals have proven susceptible to transmission (chimpanzee, rodents, cattle, felines, and mustelids), while others demonstrated resistance: dogs, horses, rabbits, and pigs (Chandler, 1971; Zlotnik and Rennie, 1965; Chandler and Fisher, 1963; Zlotnik and Rennie, 1963; Gibbs and Gajdusek, 1973; Barlow and Rennie, 1976; Kirkwood and Cunningham, 1994; Sigurdson and Miller, 2003). These natural differences in susceptibility to prion diseases can be exploited to dissect the rules governing PrP misfolding and disease. It is likely that disease susceptibility is encoded by differences in amino acid sequences that modulate conformational dynamics without a relevant impact of the cellular milieu (Vorberg et al., 2003; Vilette et al., 2001). This knowledge can be leveraged to unravel how sequence variation (genotype) impacts PrP toxicity (phenotype) (Myers et al., 2020).

Over the past few years, we and others created transgenic *Drosophila* models expressing wild-type (WT) and mutant PrP from susceptible and resistant animals: Syrian hamster, mouse, sheep, rabbit, dog and horse (Sanchez-Garcia and Fernandez-Funez, 2018; Fernandez-Funez et al., 2010, 2009; Gavin et al., 2006; Thackray et al., 2012a,b). These studies support the preservation of the intrinsic properties of each PrP when expressed in flies: WT hamster, mouse and sheep PrP are toxic in flies, whereas WT rabbit, horse and dog are not. Toxicity correlates with PrP conformational dynamics, with rabbit, horse and dog PrP showing low degrees of  misfolding and aggregation (Fernandez-Funez et al., 2010; Khan et al., 2010; Vidal et al., 2020; Otero et al., 2019; Erana et al., 2017; Fernández-Borges et al., 2017). Additionally, *Drosophila* demonstrates high sensitivity to subtle changes in the PrP sequence: hamster PrP is more toxic than mouse PrP (Fernandez-Funez et al., 2010), whereas dog and horse PrP carrying humanized mutations become toxic in progressive brain degeneration and locomotor assays (Sanchez-Garcia and Fernandez-Funez, 2018). These assays are time consuming, which dramatically narrows the utility of existing fly models. *Drosophila* is an ideal model organism for cost-effective and efficient gene discovery using robust, easy to score and sensitive assays, like in the eye. Unfortunately, existing PrP models are not toxic to the fly eye (Fernandez-Funez et al., 2017), thereby limiting their application.

To expand the utility of *Drosophila*, we examined whether PrP from other animals was more toxic. We hypothesized that human PrP is likely to be more toxic than PrP from other mammals with naturally occurring prion diseases (bovine, sheep, deer, moose). First, human prion diseases, unlike those in other animals, present with sporadic, genetic and infectious etiologies, arguing for higher structural instability of human PrP. Second, human prion diseases are heterogeneous brain disorders with different manifestations. Animal endemic prion diseases seem to have homogeneous presentations in each host. Third, these clinical differences can be attributed to diverse prion strains with specific neurotropisms, supporting the higher conformational dynamics of human PrP. Fourth, inherited prion diseases in humans are caused by >50 missense mutations, some of which introduce subtle changes (e.g. V180I, V210I). Thus, minor sequence perturbations dramatically alter human PrP dynamics. To test this idea, we generated flies expressing human PrP in a BSL3 facility to limit the risk of accumulating the transmissible protease-resistant PrP (PrP^res^) conformation. We have shown recently that flies expressing human PrP-V129 exhibit a powerful new phenotype – small and glassy eyes – that supports the heightened toxicity of human PrP (Fernandez-Funez et al., 2017). However, due to differences in construct design and expression levels, we could not directly compare the toxicity of these flies against existing models expressing rodent PrP.

Here, we described additional novel phenotypes in the brain and in a behavioral assay induced by random insertions of human PrP-V129 and -M129. We also described a new suite of comparable, isogenic transgenic flies carrying human or rodent PrPs, codon-optimized and inserted in the same attP landing site (Bischof et al., 2007). These new attP2-based PrP models elegantly demonstrate the heightened toxicity of human PrP compared to hamster and mouse PrP. As proof-of-concept for the utility of the new human PrP model, we identified intrinsic and extrinsic factors modulating its toxicity. Accumulation of misfolded PrP in the ER triggers the unfolded protein response (UPR) (Hetz et al., 2007, 2003), a complex pathway with both protective and maladaptive consequences (Hetz, 2012; Moreno et al., 2012). We describe here that PERK (officially known as EIF2AK3) and activating transcription factor 4 (ATF4) loss-of-function robustly suppressed PrP toxicity, indicating that PERK is a main driver of PrP toxicity. To gain a mechanistic understanding of the sequence-structure determinants of human PrP toxicity, we introduced three protective mutations from animals resistant to prion diseases (Sanchez-Garcia and Fernandez-Funez, 2018). D167S and N174S partially suppress human PrP toxicity, whereas N159D does not – illustrating the high structural stability of human PrP. These improved *Drosophila* models of proteinopathies provided expanded opportunities to identify the intrinsic and extrinsic factors mediating PrP toxicity, including high-throughput genetic screens and targeted amino acid replacements to determine the rules governing PrP toxicity.

## RESULTS

### Structural differences between human and rodent PrP

**Fig. 1.**
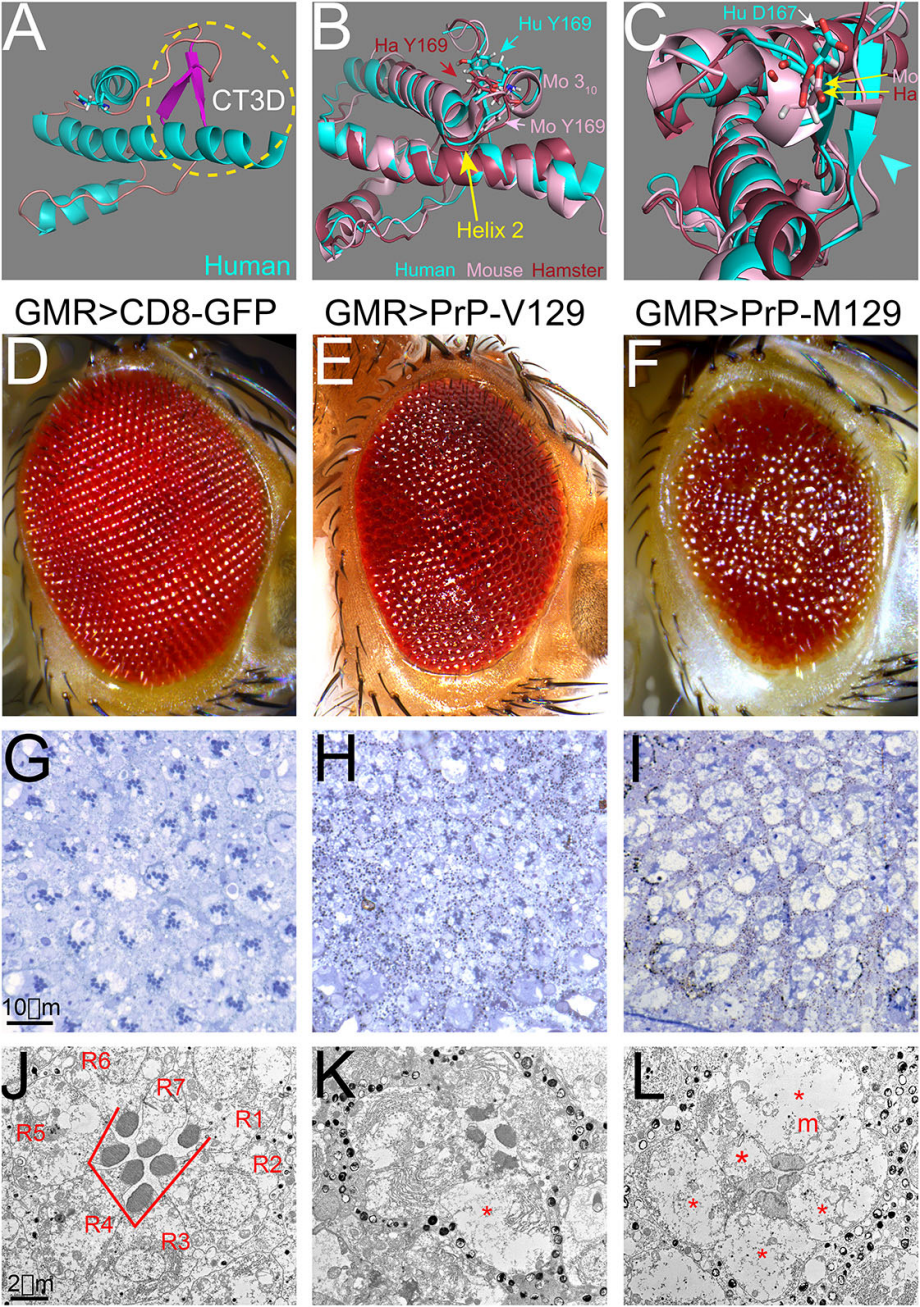
**New eye phenotypes of random human PrP lines.** (A-C) 3D visualization of the PrP globular domain. (A) Human PrP and the CT3D domain (circle). (B,C) 3D alignment of human (cyan), mouse (pink) and hamster (brown) globular domains show high conservation. Mouse has a 3_10_ turn in the loop and a longer helix 2 (C, arrow). The position of D167 and Y169 are indicated (B,C). The β-sheet has different length (C, arrowhead). (D-L) Eyes from flies expressing GFP (*GMR-Gal4*/*UAS-mCD8-GFP*) (D,G,J), human PrP-V129 (*GMR-Gal4/UAS-R-human PrP-V129*) (E,H,K), or human PrP-M129 (*GMR-Gal4/UAS-R-human PrP-M129*) (F,I,L) from random insertions at 27°C. (D-F) Micrographs of fresh eyes. Control flies and flies expressing *mCD8-GFP* exhibit highly organized eyes (D). Flies expressing human PrP-V129 or PrP-M129 display disorganized eyes (E,F). (G-I) Semithin sections of the retina. (G) Expression of GFP preserves the lattice and photoreceptors. (H) Expression of PrP-V129 results in disorganized ommatidia and loss of photoreceptors. (I) Expression of PrP-M129 results in vacuolated retina with loss of photoreceptors. (J-L) Transmission electron micrographs of single ommatidia. (J) Expression of GFP preserves seven photoreceptors (R1-R7) and rhabdomeres. (K) Expression of PrP-V129 results in partial vacuolation of photoreceptors (*), abnormal rhabdomeres, and excess of ER (arrowheads). (L) Expression of PrP-M129 results in vacuolated photoreceptors (*) and mitochondria (m) and hypochromic rhabdomeres.

The sequence alignment of the globular domain of human PrP demonstrated extensive similarity to that of hamster and mouse PrPs, albeit with minor differences (Fig. S1A). All the sequences were numbered according to human PrP. Most amino acid differences between human and rodent PrP are conservative, i.e. yielding similar chemical properties. Helix 2 and the first half of helix 3 were identical for the three sequences, whereas helix 1 displayed one amino acid difference. Most variations were concentrated in the loops and the end of helix 3. The highly variable region comprising the loop between the β-sheet and helix 2 (β2-α2 loop) forms a 3D domain with distal helix 3 (Fig. 1A). This domain is proposed to play a crucial role in PrP conversion (Telling et al., 1995; Kaneko et al., 1997). For simplicity, we termed this region the C-terminal 3D (CT3D) domain (Fig. 1A). The 3D alignment of the globular domain of human and rodent PrP (Zahn et al., 2000; Calzolai et al., 2000; James et al., 1997; Riek et al., 1996) showed overt similarity (Fig. 1B,C). Mild differences may underlie their distinct toxicity. Human PrP had a longer, i.e. more stable, β-sheet than rodent PrPs, despite perfect sequence conservation (Fig. 1C). Mouse PrP had a 3_10_ turn in the β2-α2 loop that indicates increased stability (Fig. 1B,C). Additionally, helix 2 starts at N173 in human PrP, Q172 in hamster PrP and N171 in mouse PrP, resulting in a shorter helix in human PrP (Fig. 1B, arrow). Two conserved amino acids in the loop, D167 and Y169, are more exposed in human than in mouse and hamster, creating a more open loop (Fig. 1B,C). In the surface visualization of human PrP, the side chains of D167 and E168 are perpendicular to helix 3, resulting in a positive charge (Fig. S1B,E). Most animals carry D167-Q168 in the equivalent positions (Fig. S1A), resulting in a domain that is less charged. In mouse PrP, Q168 was upward but the rest of the loop is lower (Fig. S1C,F). Interestingly, the loop in hamster PrP was lower and flatter than in human and mouse PrP, resulting in a closer interaction with helix 3 (Fig. S1D,G). Overall, these subtle structural differences suggested that human PrP is less stable than rodent PrP, which informs our hypothesis.

### New *Drosophila* eye phenotype due to random insertions of human PrP

Random insertion of codon-optimized human PrP-V129 induces a new eye phenotype not seen in flies expressing hamster PrP (Fernandez-Funez et al., 2017). We characterized here the toxicity of codon-optimized human PrP-V129 and PrP-M129 from random insertions. M/V129 is a polymorphism in human PrP, significant for the risk of variant Creutzfeldt-Jacob disease (CJD) transmission from cattle but otherwise does not impact on the causation of prion diseases (Kobayashi et al., 2015). Expression of PrP-V129 and PrP-M129 resulted in disorganized, glassy eyes (Fig. 1D-F), with PrP-M129 causing a smaller eye (Fig. 1F). Semithin sections (1 µm thick) showed that control flies display a regular arrangement of ommatidia, the visual units of the compound eye (Fig. 1G). Most ommatidia contained seven photoreceptors, recognized for the specialized photosensitive rhabdomeres in the center. Flies expressing human PrP-V129 had disorganized and vacuolated retinas (Fig. 1H). Most ommatidia contained fewer photoreceptors and their arrangement appeared disrupted. Flies expressing human PrP-M129 showed retinas with prominent disorganization and vacuolation, and few recognizable rhabdomeres (Fig. 1I). Transmission electron microscopy (TEM) showed the normal polygonal arrangement of seven photoreceptors (R1-R7) around the rhabdomeres in control flies (Fig. 1J). Flies expressing PrP-V129 showed rhabdomere loss and the remaining rhabdomeres were small and disorganized (Fig. 1K). One of the photoreceptors (*) appeared vacuolated and others contain hyperplastic endoplasmic reticulum (ER) (Fig. 1K, arrowheads). Flies expressing PrP-M129 showed few rhabdomeres and extensive vacuolation of photoreceptors (Fig. 1L, *). The rhabdomeres showed low electron density and fusions. Lastly, mitochondria appeared vacuolated with disrupted internal membranes (Fig. 1L, m). Overall, human PrP-V129 and PrP-M129 showed robust eye perturbations affecting rhabdomere differentiation and cell survival, with characteristic vacuolar degeneration that have not been described previously in flies expressing animal PrP.

### New brain phenotypes caused by random human PrP insertions

**Fig. 2.**
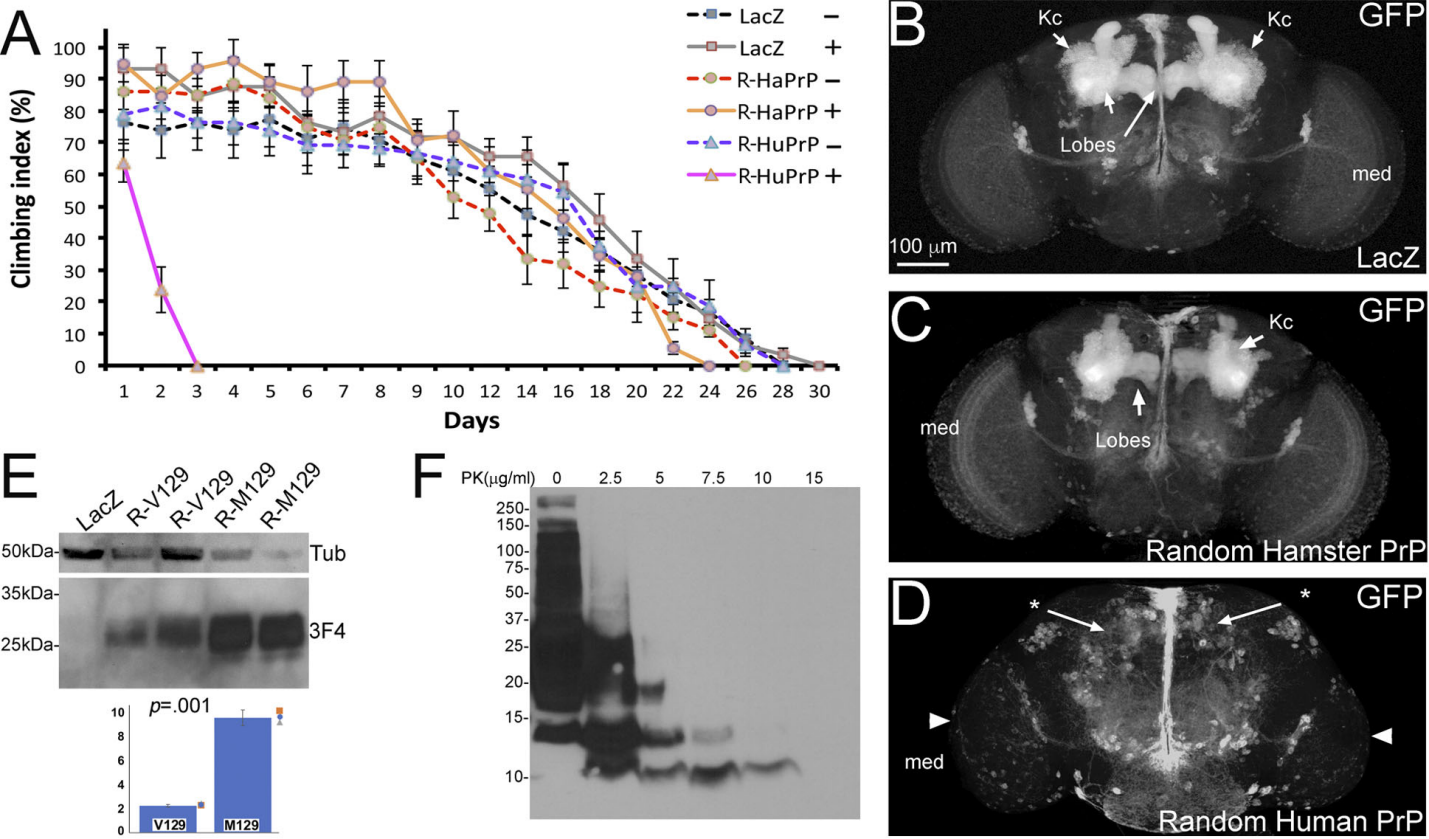
**New phenotypes induced by human PrP in *Drosophila*.** (A) Random insertions of human PrP induce aggressive locomotor dysfunction. Conditional pan-neural expression of LacZ (squares, *Elav-GS; UAS-LacZ*), hamster PrP (circles, *Elav-GS; UAS-R-HaPrP*) and human PrP (triangles, *Elav-GS; UAS-R-HuPrP-V129*). Expression was activated at day 1 (+, continuous line) or not activated (-, broken line) (*n*=2). Only flies expressing human PrP exhibit locomotor dysfunction. (B-D) Expression of human PrP in the mushroom bodies. (B) Expression of LacZ (*OK107-Gal4/UAS-mCD8-GFP/UAS-LacZ*) reveals large mushroom body (MB) clusters in 1-day-old flies, including Kenyon cell clusters (Kc) and axonal lobes. (C) Expression of hamster PrP (*OK107-Gal4/UAS-mCD8-GFP/UAS-R-HaPrP*) has no effect on the MB. (D) Expression of human PrP-V129 (*OK107-Gal4/UAS-mCD8-GFP/UAS-R-HuPrP-V129*) eliminates the mushroom bodies and results in a smaller medulla (med, arrowhead). (E) Fly homogenates expressing LacZ (lane 1), human PrP-V129 (lanes 2 and 3), or human PrP-M129 (lanes 4 and 5) in the eye from random insertions at 27°C (same genotypes as in Fig. 1). PrP-V129 and PrP-M129 display similar electrophoretic mobility (3F4 anti-PrP antibody), but PrP-M129 accumulates at levels that are over four times higher as those indicated by bar graph (*n*=3). (F) Homogenates from 10-day-old heads expressing human PrP in the eye subjected to a mild proteinase K gradient. The 10 µg/ml treatment degraded most PrP except for a 10 kDa fragment. The 15 µm/ml digestion has no detectable PrP.

Flies constitutively expressing human PrP under the control of the pan-neural driver *Elav-Gal4* showed 100% lethality at 25°C. In contrast, flies expressing hamster PrP under the same conditions were 100% viable. To bypass this developmental toxicity, we used the *Elav-GeneSwitch* driver (*Elav-GS*), a conditional Gal4 activated by the steroid hormone mifepristone (RU486) (Roman et al., 2001). We combined LacZ (negative control), hamster PrP and human PrP with *Elav-GS*, and grew the flies in medium lacking RU486 to allow development in the absence of PrP expression. Then, we placed newly eclosed adult flies in vials with or without RU486 at 28°C (Day 0) and subjected them to climbing assays. Control experiments (−RU486) showed similar climbing ability in flies carrying LacZ, hamster PrP or human PrP constructs (Fig. 2A). Flies expressing LacZ (+RU486) reached a 50% climbing index by day 16 and climbed until day 28 (Fig. 2A). Flies expressing hamster PrP (+RU486) reached a 50% climbing index at day 14 and climbed until day 26 (Fig. 2A). However, flies expressing human PrP (+RU486) reached a 50% climbing index by day 1.5 and only climbed for 3 days (Fig. 2A). Differences among groups were analyzed by fitting a kinetics model and calculating area under each curve, indicating significant differences for the HuPrP+RU group (Fig. S2, Tables S1 and S2). The fast progression of the locomotor dysfunction illustrated the high toxicity of human PrP.

We next monitored the impact of human PrP on a brain center not crucial for survival. Mushroom bodies are a well-known brain region involved in higher neural processing in insects, including memory and learning (Davis, 2005; Tanaka et al., 2008). They are two symmetric centers comprising 2500 neurons each, with the cell bodies in the posterior brain and the axonal projections extending to the front. Expression of LacZ or hamster PrP in mushroom body neurons (*OK107-Gal4*) showed robust architecture at day 1 post eclosion (Fig. 2B,C). Notably, flies expressing human PrP from at least 12 brains lacked recognizable mushroom body structures (Fig. 2D). The optic lobes were smaller due to weak expression of *OK107-Gal4* (Fig. 2D, arrowheads). Overall, these new phenotypes in the brain supported our hypothesis that human PrP is more toxic than rodent PrPs. However, these phenotypes are not directly comparable since only human PrP was codon-optimized and each construct is subjected to different position effects.

### Protein analysis of randomly inserted human PrP

Homogenates from the heads of 1-day-old flies expressing LacZ (negative control), human PrP-V129 or PrP-M129 in the eye were subjected to western blotting with the anti-PrP antibody 3F4. Levels of PrP-M129 were approximately 4-fold higher than those for PrP-V129, possibly explaining the difference in eye phenotype (Fig. 2E). The different expression level exemplifies the problem with random insertions. We next determined whether human PrP spontaneously accumulates protease resistant PrP conformations in *Drosophila*. Transmissible prions contain PrP^res^, which is resistant to denaturing agents and proteinase-K digestion (20 µg/ml for 1 h at 37°C) (McKinley et al. (1983). Digestion with proteinase K of PrP^res^ resulted in a diagnostic proteinase K-resistant and transmissible core fragment of ∼20 kDa. We expressed human PrP in the eye, kept the flies for 10 days, homogenized their heads and subjected them to a mild proteinase K gradient (2.5-15 µg/ml for 30 min at 25°C) (Fig. 2F). 5 µg/ml proteinase K eliminated full-length PrP but left fragments below 20 kDa. Proteinase K levels of 7.5 and 10 µg/ml eliminated almost all the signal, except for small fragments of ∼12 and 10 kDa. Finally, 15 µg/ml proteinase K eliminated all PrP signal. Thus, digestion under mild proteinase K conditions demonstrated that human PrP does not spontaneously form PrP^res^ in *Drosophila*.

### New human and rodent PrP constructs: codon-optimized attP2 lines

**Fig. 3.**
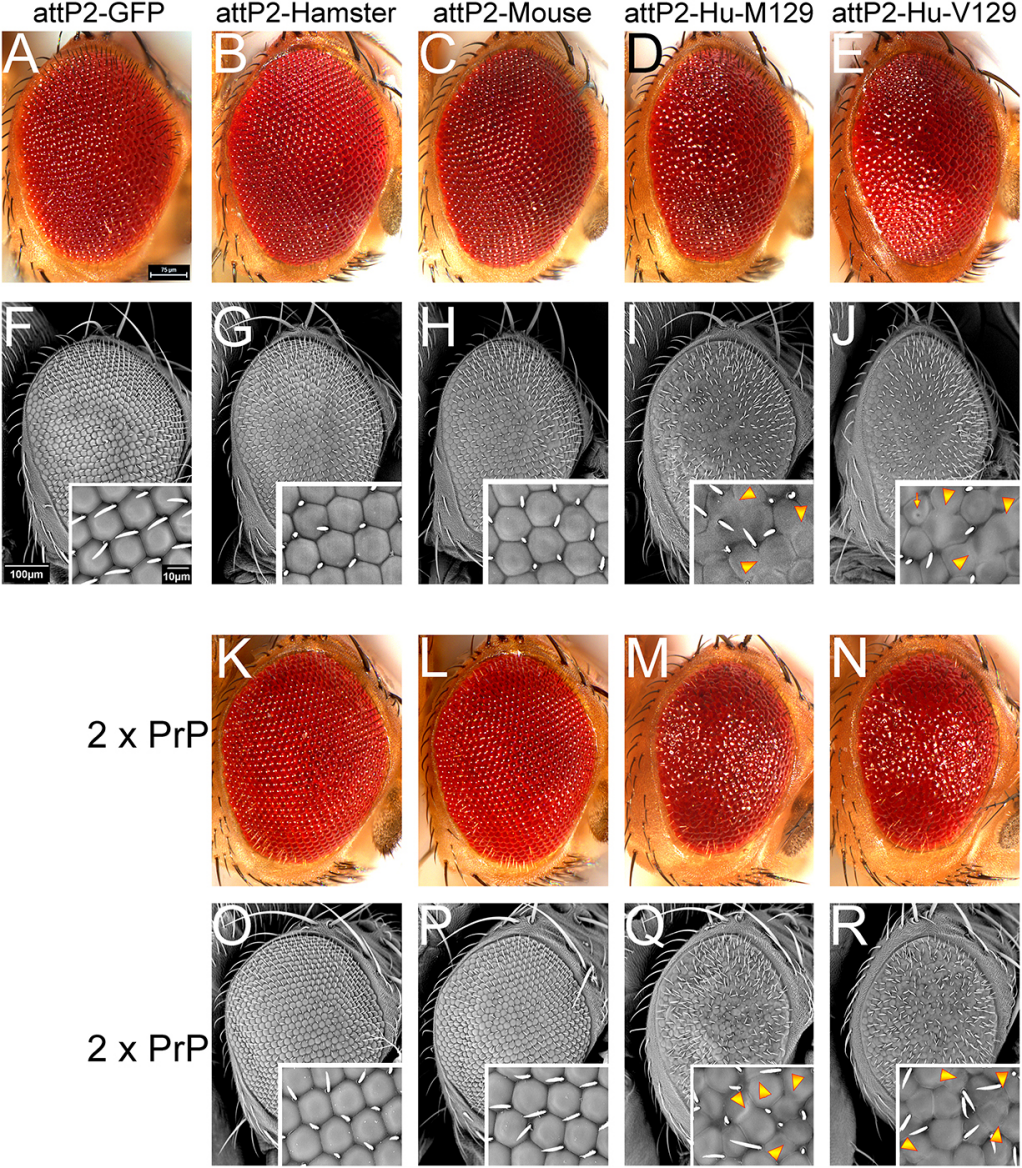
**Human PrP-attP2 constructs are more toxic than rodent PrP-attP2.** Fresh eyes and scanning electron micrographs of eyes expressing the attP2-PrP constructs. A-J, one copy of PrP constructs; K-R, two copies. (A,F) Control eyes from flies expressing mCD8-GFP-attP2 (*GMR-Gal4/UAS-mCD8-GFP-attP2*) display a highly organized lattice. Flies expressing PrP from hamster (B,G) (*GMR-Gal4/UAS-hamster PrP-attP2*) or mouse (C,H) (*GMR-Gal4/UAS-mouse PrP-attP2*) show normal eyes. (D,E,I,J) Flies expressing human PrP (*GMR-Gal4/UAS-human PrP-M129-attP2* or *GMR-Gal4/UAS-human PrP-V129-attP2*) display mildly disorganized eyes. (K-R) Flies expressing two copies of PrP (2 x PrP) are (*GMR-Gal4/+; UAS-PrP-attP2/UAS-PrP-attP2*). Flies expressing two copies of hamster (K,O) or mouse (L,P) PrP display well organized eyes. (M,N,Q,R) Flies expressing human PrP-M129 (M,Q) or PrP-V129 (N,R) display smaller and glassy eyes.

To directly compare the toxicity of human and rodent PrP, we generated a comparable suite of PrP constructs. Constructs were first codon-optimized for *Drosophila* expression and then inserted in the same molecularly defined locus, i.e. the strong attP2 landing site we have used previously (Bischof et al., 2007; Moore et al., 2018). These new constructs enabled comparative studies in which any differences in toxicity can be directly attributed to sequence differences. For human PrP, we generated the two natural polymorphisms (M129 and V129) to examine their behavior when expressed from comparable insertions. Flies expressing mouse or hamster PrP-attP2 had normal eyes, similar to those of control flies (Fig. 3A-C,F-H). Flies expressing human PrP-M129-attP2 or PrP-V129-attP2 showed mild disorganization of the eye (Fig. 3D,E,I,J). Magnification showed poor differentiation of ommatidia with multiple fusions (Fig. 3I,J, arrowheads). The eye phenotype of the two human PrP-attP2 lines was, as expected, weaker than those from random insertions (Fig. 1) due to lower expression levels.

Since the human attP2-PrP constructs induce mild eye phenotypes, it could be argued that rodent PrPs cause detectable phenotypes by pushing their expression. To test this, we generated flies carrying two copies of the PrP-attP2 constructs with one copy of *GMR-Gal4*. Flies expressing 2x mouse or hamster PrP-attP2 still exhibited normal eyes (Fig. 3K,L,O,P). In contrast, flies expressing 2x human PrP-attP2 exhibited small and very disorganized eyes (Fig. 3M,N,Q,R). The ommatidia had abnormal shapes and appeared fused (Fig. 3Q,R, insets). Thus, doubling the expression of PrP resulted in qualitative differences in eye toxicity between rodent and human PrP, which supports the heightened toxicity of human PrP.

### mRNA expression analyses of the new attP2 PrP lines

**Fig. 4.**
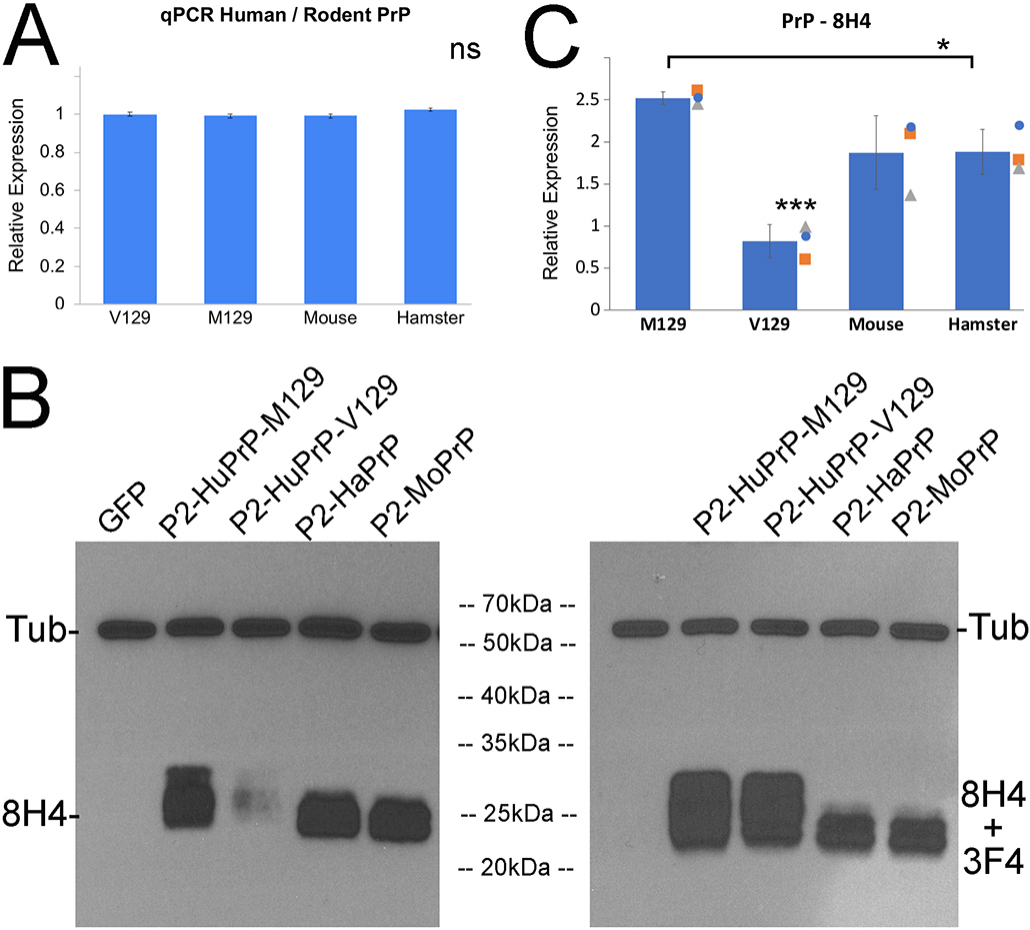
**Human and rodent PrP undergo different biogenesis.** (A-C) Expression of attP-based constructs in the eye (same genotypes as in Fig. 3). (A) Levels of PrP mRNA by qPCR are identical for all PrP construct (2 independent experiments). (B) Western blotting. Left: detection of PrP from fly heads expressing the indicated construct in the eyes by using 8H4 anti-PrP and anti-tubulin antibodies. The electrophoretic pattern of human PrP-M129 is different to rodent PrP. PrP-V129 is weakly stained by 8H4 antibody. Right: same membrane serially incubated with antibodies 8H4 and 3F4, showing normal levels of human PrP-V129. (C) Quantification of PrP signals with 8H4 antibody (*n*=3). *P<0.05; ***P<0.001.

We next examined mRNA expression levels for the new attP2-based lines by quantitative RT-PCR (qPCR). We generated homogenates from flies expressing attP2-PrP in the eye as described above, followed by qPCR. The same primers were used for human PrP-M129 and PrP-V129, but hamster and mouse PrP each required unique primers because of small sequence differences. After normalization to *G3PDH* mRNA levels, all constructs showed identical expression levels (Fig. 4A), consistent with the shared landing site at attP2.

Next, we analyzed the new PrP lines for differences in the relative accumulation of isoforms. PrP has two facultative *N*-glycosylation sites and the relative usage of these two sites depends on their availability. We generated homogenates from flies expressing mCD8-GFP-attP2 or PrP-attP2 in the eye as described above. We first used the 8H4 anti-PrP antibody that binds both human and rodent PrP. Antibody 8H4 revealed strong reactivity against human PrP-M129, hamster and mouse PrP but only weakly stained human PrP-V129 (Fig. 4B, left panel). Note that all lanes were loaded equally, as indicated by the tubulin loading control. Quantification of three biological replicates showed that human PrP-M129 accumulates at levels higher than those of hamster PrP (*P*=0.048) and mouse PrP (*P*=0.121), although mouse PrP shows more variability (Fig. 4C). PrP-V129 levels are significantly lower than those of all other samples. This finding was consistent over multiple replicates. Compared with PrP-M129, it is unlikely that PrP-V129 is expressed at very low levels as both PrPs induce similar eye phenotypes (Fig. 3). One possible explanation is that the epitope of antibody 8H4 detects a conformational difference between PrP-M129 and PrP-V129 polymorphisms. Unfortunately, only few antibodies detect conserved epitopes in human, hamster as well as mouse PrP, and – even less likely – with the same affinity. We serially incubated the same membrane with antibodies 8H4 and 3F4, neither of which recognizes mouse PrP. The combination of antibodies 8H4+3F4 showed similar signal intensity and electrophoretic pattern for PrP-V129 and PrP-M129 (Fig. 4B, right panel). Both human PrPs presented a strong diglycosylated isoform that is lacking in hamster and mouse PrP, revealing differences in biogenesis.

### Subcellular distribution of the new attP2 PrP lines

**Fig. 5.**
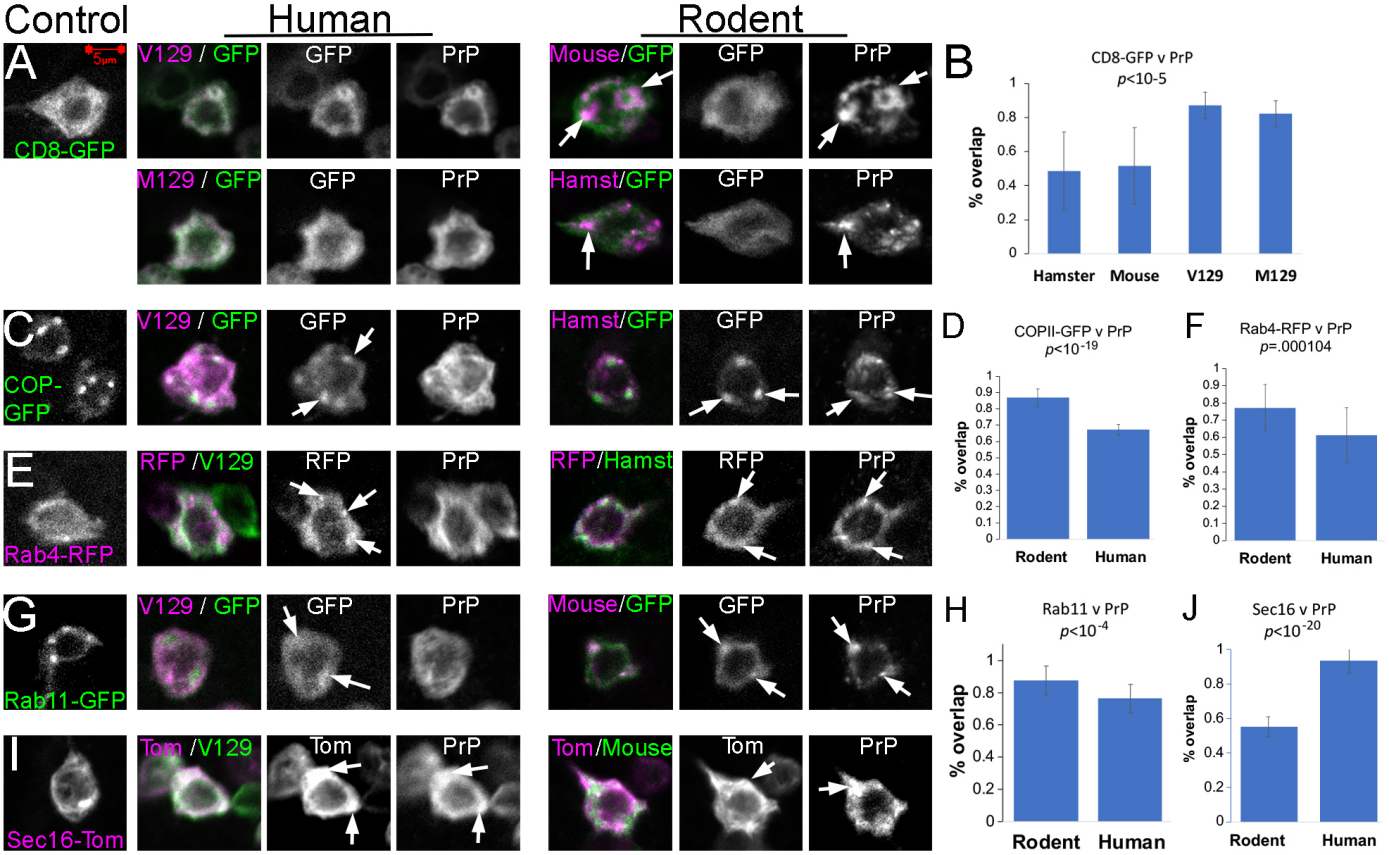
**Subcellular distribution of rodent and human PrP.** (D-F) Distribution of human PrP in interneurons of the larval ventral ganglion (*OK107-Gal4/UAS-attP2-PrP/UAS-GFP-X*). (A) Co-expression of *UAS-mCD8-GFP* and *UAS-LacZ* (control), human PrP (*UAS-attP2-HuPrP-V129 or UAS-attP2-HuPrP-M129*) or rodent PrP (*UAS-attP2-hamster PrP or UAS-attP2-mouse PrP*). Human PrP shows diffuse expression and rodent PrP has punctate distribution. (B) Fraction of mCD8-GFP and PrP overlap. (C) Co-expression of *UAS-COPII-GFP* and *UAS-LacZ* (control), human PrP (*UAS-attP2-HuPrP-V129*) or rodent PrP (*UAS-attP2-hamster PrP*). (D) Fraction of COPII-GFP and PrP overlap. (E) Co-expression of *UAS-Rab4-RFP* and LacZ (control), human PrP (*UAS-attP2-HuPrP-V129*) or rodent PrP (*UAS-attP2-hamster PrP*). (F) Fraction of Rab4-RFP and PrP overlap. (G) Co-expression of *UAS-Rab11-GFP* and LacZ (control), human PrP (*UAS-attP2-HuPrP-V129*) or rodent PrP (*UAS-attP2-mouse PrP*). (H) Fraction of Rab11-GFP and PrP overlap. I, Co-expression of *UAS-Sec16-Tomato* and LacZ (control), human PrP (*UAS-attP2-HuPrP-V129*) or rodent PrP (*UAS-attP2-mouse PrP*). (H) Fraction of *Sec16-Tomato* and PrP overlap. All images were collected at the same magnification; scale bar shown in A is applicable to all panels. Data were created from 12-15 neurons from observations replicated in more than six brains.

We next examined the subcellular distribution of rodent and human PrP to examine their transition through the secretory pathway. We co-expressed PrP-attP2 together with reporter constructs in interneurons of the larval ventral ganglion (*OK107-Gal4*). mCD8-GFP was used to label the plasma membrane but also to stain intracellular compartments of the secretory pathway (Fig. 5A). Human PrP shows diffuse intracellular distribution and extensive overlap with mCD8-GFP (Fig. 5A,B). Both rodent PrPs showed punctate intracellular distribution (Fig. 5A) (Fernandez-Funez et al., 2010, 2009) with a 50% overlap with mCD8-GFP (Fig. 5B). γCOPII-GFP labeled 3-5 vesicles connecting the ER with the Golgi apparatus in small interneurons and more in larger neurons (Fig. 5C). Human PrP overlapped with γCOPII-GFP during its transit to the ER, but rodent PrP showed a larger overlap (Fig. 5C,D). Rab4-RFP (early endosomes) showed a few puncta per cell and some overlap with human PrP (Fig. 5E). Rodent PrP showed more overlap with the Rab11 puncta (Fig. 5E,F). Rab11 (recycling endosomes) also accumulated in a few puncta per interneuron in controls (Fig. 5G). Human PrP showed partial overlap with Rab11 but rodent PrP showed higher overlap (Fig. 5G,H). Last, Sec16-Tomato (used to stain secretory vesicles) showed diffuse expression and intracellular distribution, with a large vesicle close to the membrane (Fig. 5I). Human PrP showed almost complete overlap with Sec16, whereas rodent PrP showed ∼50% overlap (Fig. 5I,J). Overall, these analyses show significant differences in the subcellular distribution of human and rodent PrP. For unknown reasons, rodent PrP is retained in several compartments of the secretory pathway whereas human PrP seems to have a smoother transition without retention in any specific vesicle.

### Extrinsic modifiers of PrP toxicity: interaction between human PrP and the amyloid-β peptide

We further tested the differences between human and rodent PrP by examining genetic interactions with other factors. Multiple reports support direct interaction between PrP and the amyloid-β42 (Aβ42) peptide in biochemical assays (Laurén et al., 2009; Chen et al., 2010; Zou et al., 2011; Gimbel et al., 2010; Gunther and Strittmatter, 2010; Balducci et al., 2010). PrP might be required for the manifestation of Aβ phenotypes in brain neurons in mouse models, suggesting a functional link between Alzheimer's and prion diseases. The new PrP-attP2 lines allowed us to test whether human and rodent PrP show similar functional interactions with Aβ42. Since high expression of Aβ42 yields robust eye phenotypes at 27°C (Casas-Tinto et al., 2011), we examined interactions with PrP at 25°C. As shown above, flies expressing hamster and mouse PrP-attP2 have normal eyes (Fig. S3A-C), whereas expression of human PrP-M129-attP2 or PrP-V129-attP2 resulted in subtle disorganization (Fig. S3D,E). Co-expression of Aβ42 and GFP results in moderately disorganized eyes with a few black spots (Fig. S3F). Co-expression of hamster and mouse PrP with Aβ42 results in eyes similar to those of control flies (Fig. S3G,H). Remarkably, co-expression of human PrP-M129 or PrP-V129 with Aβ42 results in small and highly disorganized (glassy) eyes (Fig. S3I,J), demonstrating a specific functional interaction between human PrP and Aβ42.

### Extrinsic modifiers of PrP toxicity: the UPR

**Fig. 6.**
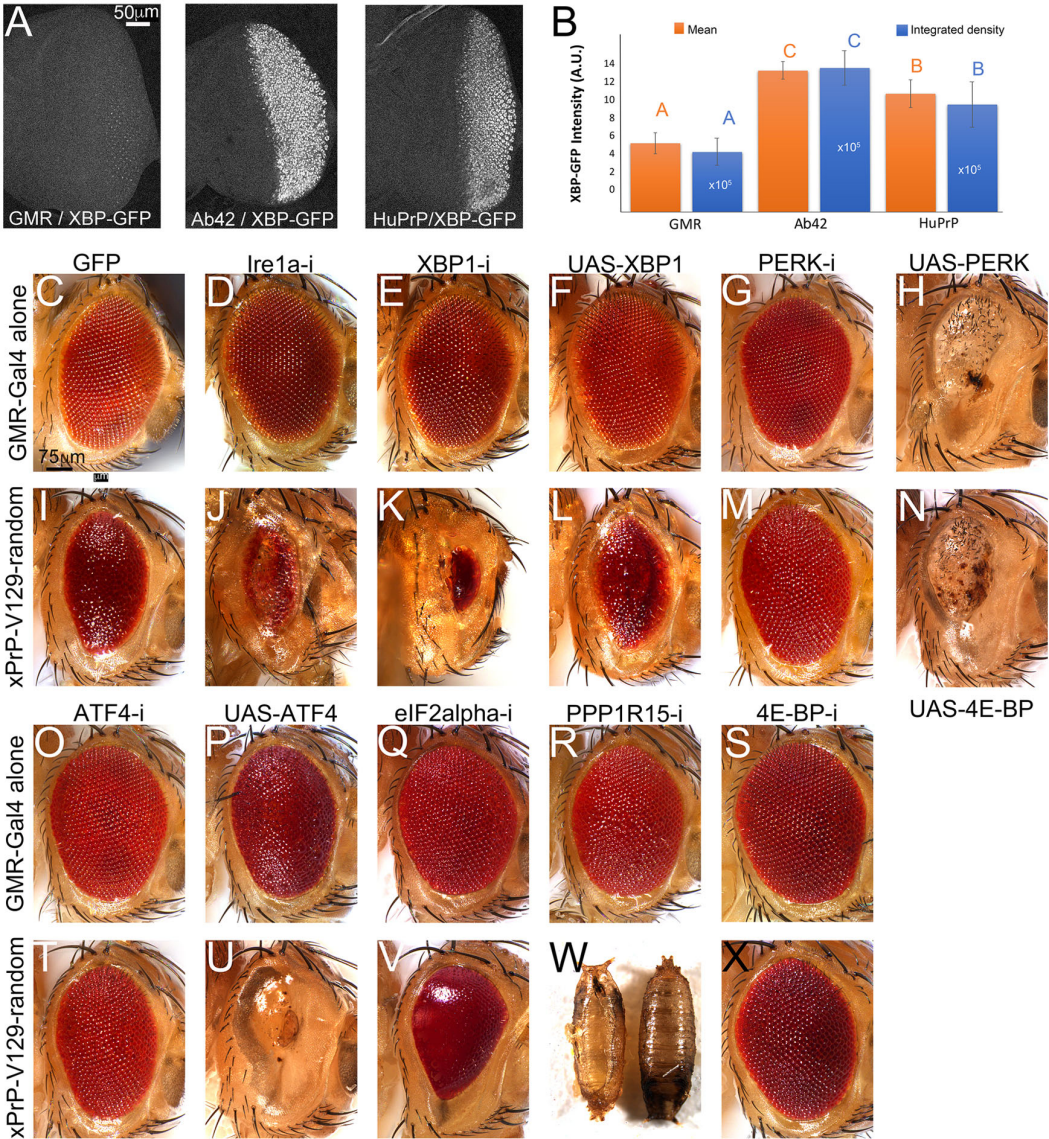
**Silencing PERK and ATF4 suppresses human PrP toxicity.** (A,B) Human PrP activates the XBP-GFP sensor (*GMR-Gal4/UAS-XBP-GFP*). Both human PrP (*UAS-R-Human PrP-V129*) and Aβ42 (*UAS-R-Aβ42*) activate XBP-GFP above the endogenous levels, but Aβ42 is stronger. (B) Mean signal (orange) and integrated intensity (blue) are analyzed independently. Scale for the integrated density is ×10^5^. All differences are statistically significant as indicated by non-overlapping letters from ANOVA. (C-Z) Micrographs of fresh eyes expressing UPR alleles alone or in combination with human PrP in the eye at 27°C. (C,I) Control flies expressing mCD8-GFP (C, *GMR-Gal4/UAS-mCD8-GFP-attP2*) or co-expressing PrP (I, *GMR-Gal4/UAS-mCD8-GFP-attP2/UAS-R-human PrP-V129*). (D-F) Flies carrying Ire1α branch alleles (D, *GMR-Gal4/UAS-Ire1α-RNAi*), XBP1-RNAi (E, *GMR-Gal4/UAS-XBP1-RNAi*) or XBP1 (F, *GMR-Gal4/UAS-XBP1*) alone exhibit normal eyes. (J,K) Flies co-expressing PrP and Ire1α-RNAi (J, *GMR-Gal4/UAS-Ire1α-RNAi/UAS-R-human PrP-V129*) or XBP1-RNAi (K, *GMR-Gal4/UAS-XBP1-RNAi/UAS-R-human PrP-V129*) exhibit small eyes. (L) Flies co-expressing PrP and XBP1 (*GMR-Gal4/UAS-XBP1/UAS-R-human PrP-V129*) show no change. (G,O) Flies expressing PERK-RNAi (G, *GMR-Gal4/UAS-PERK-RNAi*) or ATF4-RNAi (O, *GMR-Gal4/UAS-ATF4-RNAi*) alone exhibit normal eyes. (M,U) Flies co-expressing PrP and PERK-RNAi (M, *GMR-Gal4/UAS-PEK-RNAi/UAS-R-human PrP-V129*) or ATF4-RNAi (U, *GMR-Gal4/UAS-ATF4-RNAi/UAS-R-human PrP-V129*) exhibit large eyes. (H,N) Flies expressing PERK alone (H, *GMR-Gal4/UAS-PERK*) or co-expressing PrP (N, *GMR-Gal4/UAS-PERK/UAS-R-human PrP-V129*) have small eyes. (P) Flies expressing ATF4 (*GMR-Gal4/UAS-ATF4*) have mildly disorganized eyes. (V) Flies co-expressing PrP and ATF4 (*GMR-Gal4/UAS-ATF4/UAS-R-human PrP-V129*) show small eyes. (Q) Flies expressing eIF2α-RNAi (*GMR-Gal4/UAS-eIF2α-RNAi*) exhibit mildly disorganized eyes. (W) Flies co-expressing PrP and eIF2α-RNAi (*GMR-Gal4/UAS-eIF2α-RNAi/UAS-R-human PrP-V129*) exhibit highly disorganized and mildly depigmented eyes. (R) Flies expressing PPP1R15-RNAi (*GMR-Gal4/UAS-PPP1R15-RNAi*) show mildly disorganized eyes. (X) Flies co-expressing PrP and PPP1R15-RNAi (*GMR-Gal4/UAS-PPP1R15-RNAi/UAS-R-human PrP-V129*) results in pupal lethality. (S,Y) Flies expressing 4E-BP-RNAi alone (S, *GMR-Gal4/UAS-4E-BP-RNAi*) show normal eyes; co-expressing PrP (Y, *GMR-Gal4/UAS-4E-BP-RNAi/UAS-R-human PrP-V129*) suppresses toxicity. (T,X) Flies expressing 4E-BP alone (T, *GMR-Gal4/UAS-4E-BP*) show normal eyes and co-expressing PrP (*GMR-Gal4/UAS-4E-BP/UAS-R-human PrP-V129*) enhances toxicity. Each observation was independently replicated at least three times.

One of the best-understood mechanisms mediating the toxicity of PrP is the accumulation of misfolded conformations in the ER, which overwhelm the folding capacity of the ER, cause ER stress and activate the UPR (Hetz et al., 2005; Moreno et al., 2012). The UPR encompasses the coordinated activity of three ER membrane-anchored sensors, i.e. PERK, Ire1α (officially known as Ire1 in *Drosophila* and ERN1 in humans) and ATF6 (Fig. S4). An increase in misfolded protein load in the ER activates the sensors and their downstream effectors. Activation of the Ire1α branch results in splicing of a 24-nt intron in the X-box binding protein 1 (XBP1) that activates XBP1s (Fig. S4). We have shown previously that Aβ42 activates the XBP1-GFP sensor (Fig. 6A) (Casas-Tinto et al., 2011; Ryoo et al., 2007). Expression of human PrP-V129 also activates XBP-GFP at levels that are significantly lower than those for Aβ42 (Fig. 6B; Table S4). In line with this, silencing of Ire1α or XBP1 in flies expressing human PrP resulted in very small eyes (Fig. 6I-K) despite these alleles not having an effect on their own (Fig. 6C-E; Table S4). These loss-of-function results reveal the protective role of Ire1α and XBP1 in PrP toxicity.

The PERK branch is the most complex of the three branches (including Ire1 and ATF6) of the UPR because it mediates both protective and maladaptive responses (Fig. S4). Activated PERK phosphorylates eIF2α and prevents the interaction of the eIF2 complex with the ribosome, resulting in global translation inhibition and resolution of acute ER stress. Yet, chronic ER stress can result in cell death by blocking translation. To resolve acute ER stress, unconventional translation of ATF4 results in the transcriptional regulation of stress response genes and the PPP1R15 phosphatase (GADD34 in mammals). PPP1R15 dephosphorylates eIF2α to restore translation. In flies, PPP1R15 is activated by eIF2α-independent translation, like ATF4, and is not downstream of ATF4 (Malzer et al., 2013). We next examined the consequence of modulating PERK and ATF4 activity on the toxicity of human PrP. Silencing either PERK or ATF4 alone has no effect in the eye (Fig. 6G,O). Remarkably, silencing PERK or ATF4 robustly suppressed PrP toxicity in the eye (Fig. 6M,T). We validated these results by using multiple lines of RNA interference (RNAi), i.e. *PEK^KK100348^*, *PEK^HMJ02063^*, *PEK^GL00030^*, *ATF4^KK111018^* and *ATF4^JF02007^* (Table S4). PERK overexpression in the eye alone or together with PrP was mostly pupal lethal but adult escapers showed very small eyes (Fig. 6H,N), supporting a key function of PERK in eye development (Malzer et al., 2010). Overexpression of ATF4 alone resulted in slightly disorganized eyes (Fig. 6P) but overexpression of ATF4 and PrP resulted in very small and glassy eyes (Fig. 6U). Silencing eIF2α alone resulted in slight eye disorganization (Fig. 6Q) and enhanced the toxicity of PrP, resulting in smaller more disorganized eyes (Fig. 6V). Last, silencing of PPP1R15 alone resulted in slightly disorganized eyes (Fig. 6R) but caused synthetic pupal lethality with PrP using two different alleles, i.e. *PPP1R15^KK104106^* and *PPP1R15^HMS00811^* (Fig. 6W). This is consistent with a significant increase in the levels of phosphorylated eIF2α (phospho-eIF2α) and inhibition of protein translation. These observations indicate that phosphorylation of eIF2α is a main driver of PrP toxicity in flies.

The robust suppression of PrP toxicity by ATF4-RNAi suggests that additional downstream effectors of ATF4 contribute to the protective activity. Recent studies have identified the 4E-binding protein (4E-BP, officially known as EIF4EBP2 in humans and Thor in *Drosophila*) as an ATF4 transcriptional target (Kim et al., 2020; Malzer et al., 2018; Kang et al., 2017; Vasudevan et al., 2017). Interestingly, 4E-BP binds eIF4E and prevents the assembly of the eIF4F complex, which is crucial for the entry of capped mRNAs into the ribosomal small subunit. Silencing 4E-BP alone has no deleterious effect in the eye (Fig. 6S) but robustly suppresses PrP toxicity (Fig. 6X). Flies only overexpressing 4E-BP showed no× significant changes (Fig. 6T) and only mildly enhanced PrP toxicity (Fig. 6Z; Table S4). These results suggest that silencing 4E-BP mediates the protective activity of ATF4, providing a second but redundant mechanism to block translation during ER stress.

### Intrinsic mediators of toxicity: protective substitutions from animals resistant to prion diseases

**Fig. 7.**
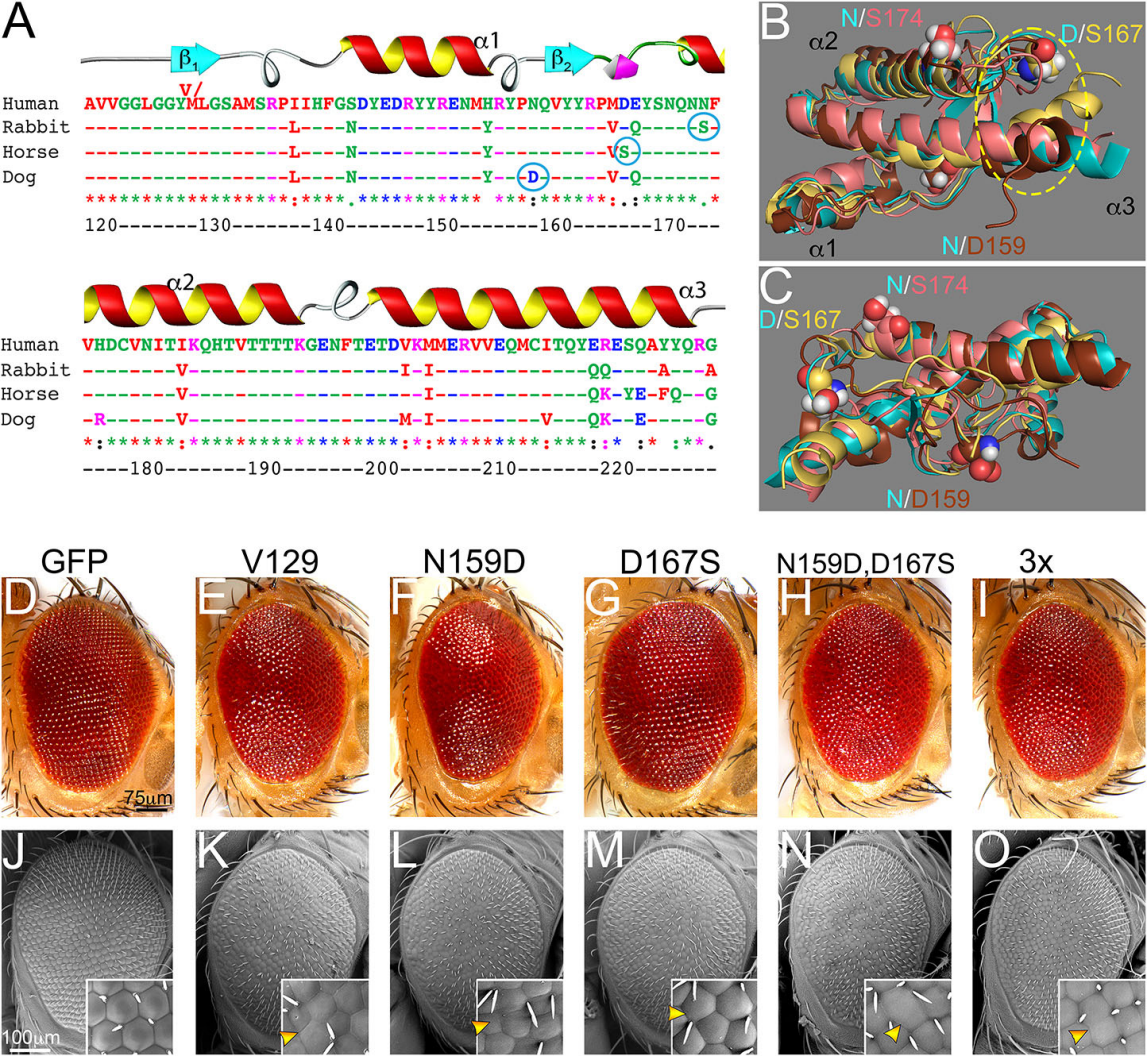
**D167S is protective in human PrP.** (A) Sequence alignment of the globular domain of human, dog, horse, and rabbit PrP. Amino acid numbering corresponds to human PrP. Candidate protective residues are circled. (B,C) 3D alignment of the globular domain of human (cyan), dog (brown), horse (yellow), and rabbit (salmon) PrP. The position of residues 159, 167and 174 is indicated. (D-O) Micrographs of fresh eyes and scanning electron microscope from control flies or flies expressing human PrP-attP2. (D,J) Control flies (*GMR-Gal4/UAS-mCD8-GFP-attP2*) show large, organized eyes. (E,K) Flies expressing human PrP-WT (*GMR-Gal4/UAS-human PrP-V129-attP2*) show disorganized, glassy eyes with ommatidia fusions (arrowhead). (F,L) Flies expressing PrP-N159D (*GMR-Gal4/UAS-human PrP-N159D-attP2*) show glassy eyes with abnormal ommatidia (arrowhead). (G,M) Flies expressing PrP-D167S (*GMR-Gal4/UAS-human PrP-D167S-attP2*) show improved eye organization (arrowhead). (H,I,N,O) Flies expressing the 2x mutant PrP (*GMR-Gal4/UAS-human PrP-N159D-D167S-attP2*) or the 3x mutant (*GMR-Gal4/UAS-human PrP-N159D-D167S-N174S-attP2*) show partially rescued eye organization but abnormal ommatidia are visible (arrowheads). Each observation was independently replicated at least three times.

Several animals are recognized for their high natural resistance to prion diseases, including dogs, horses, rabbits and pigs (Kirkwood and Cunningham, 1994; Espinosa et al., 2020; Vidal et al., 2020; Chianini et al., 2012; Bian et al., 2017). Sequence analyses shows multiple differences between PrP of these animals and that of humans; although it is unclear which substitutions are protective and which are neutral (Fig. 7A). Structural studies identified residues that have been proposed to mediate the stability of resistant PrPs: D159 in dog, S167 in horse and S174 in rabbit and pig (Myers et al., 2020; Khan et al., 2010; Pérez et al., 2010; Lysek et al., 2005). Two of these residues are in the CT3D, and D159 can impact the CT3D from a short distance (Fig. 7A). The 3D alignment of human, dog, horse, and rabbit PrP (Fig. 7B,C) shows high overall conservation. Relevant differences include the length of the β-sheet and helix, and the CT3D domain (Fig. 7B,C). However, no clear structure-function correlation exists currently. We, therefore, hypothesize that these three residues impact the dynamics of the CT3D domain in their corresponding PrPs and are responsible for the high toxicity of human PrP compared to dog, rabbit, and horse PrP.

### *In vivo* activity of protective substitutions: eye phenotype

We have previously examined the consequence of introducing the equivalent amino acid substitution from human PrP into dog, horse and rabbit PrP. Dog PrP-D159N and horse PrP-S167D become toxic in the *Drosophila* brain neurons, whereas rabbit PrP-S174N has no effect (Sanchez-Garcia and Fernandez-Funez, 2018). To examine the mechanisms mediating human PrP toxicity, we next introduced the three protective residues from dog, horse and rabbit PrP into human PrP-V129. We introduced N159D or D167S alone, together (2x-N159D-D167S) or in combination with N174S (3x-N159D-D167S-N174S). The N174S substitution alone is described elsewhere together with Y225A (R.M.M., Aliciarose John, Weiguanliu Zhang, Wen-Quan Zou, Alessandro Cembran and P.F.F., unpublished observations). We generated transgenic flies by using the methods described above (codon-optimization and insertion into the attP2 landing site in the human PrP-V129 backbone).

Flies expressing human PrP-V129-attP2 in the eye at 27°C exhibited slightly smaller and moderately disorganized eyes as those shown before (Fig. 7D,E,J,K; Table S5). Flies expressing human PrP-N159D-attP2 showed eyes similar to those in flies that expressed PrP-V129 (Fig. 7F,L; Table S5). Flies expressing human PrP-D167S-attP2 exhibited larger and better organized eyes than those expressing PrP-V129 (Fig. 7G,M; Table S5). High magnification showed more definition of ommatidia, although they are abnormal (Fig. 7M, inset). Flies expressing the 2x and the 3x mutants exhibited similar organization to D167S alone (Fig. 7H,I,N,O; Table S5), indicating no cooperative activity. Overall, these experiments showed that N159D alone is not protective in the context of human PrP, whereas the reciprocal substitution in dog PrP is toxic. D167S is partially protective but showed no cooperativity with N159D and N174S. This preliminary characterization in the eye is useful in order to move into more sensitive and quantitative assays in brain neurons.

### *In vivo* activity of protective substitutions: degeneration of brain neurons

**Fig. 8.**
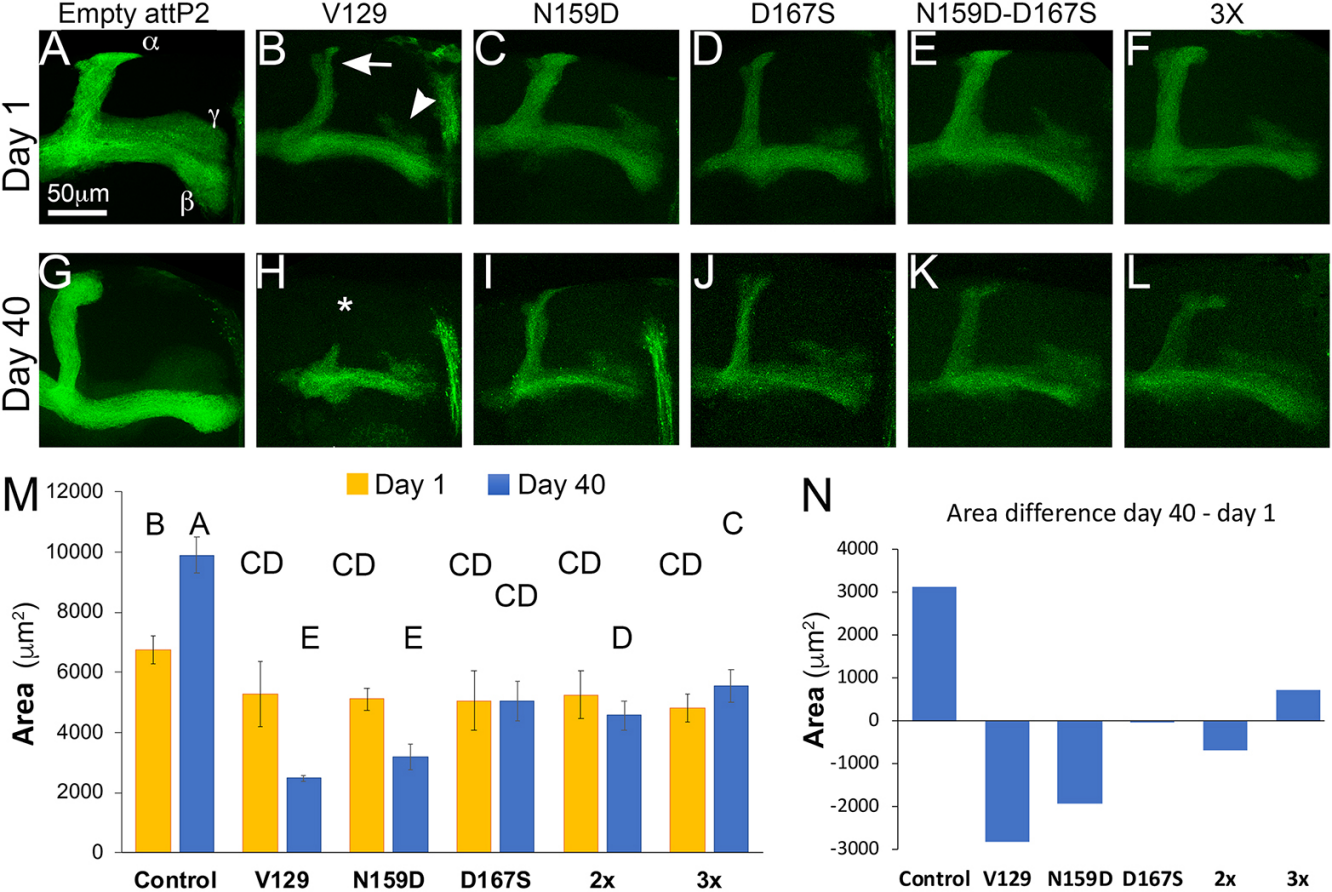
**Analysis of protective mutations in brain neurons.** (A-L) Micrographs of mushroom body axonal projections at days 1 (A-F) or 40 (G-L). (A,G) 1- and 40-day-old flies carrying an empty attP2 site (*OK107-Gal4/UAS-mCD8-GFP/attP2*) show robust mushroom body axonal projections. The α, β, and γ lobes are indicated. 40-day-old flies show an increase in projection surface. (B,H) 1- and 40-day-old flies expressing human PrP-WT (*OK107-Gal4/UAS-mCD8-GFP/UAS-human PrP-V129-attP2*) show thin projections at day 1 and significant degeneration by day 40 (H, *). (C,I) 1- and 40-day-old flies expressing PrP-N159D (*OK107-Gal4/UAS-mCD8-GFP/UAS-human PrP-N159D-attP2*) show small projections at day 1 that continue to degenerate during aging. (D,J) 1- and 40-day-old flies expressing PrP-D167S (*OK107-Gal4/UAS-mCD8-GFP/UAS-human PrP-D167S-attP2*) show small projections at day 1 but slower degeneration. (E,F,K,L) 1- and 40-day-old flies expressing the 2X (*OK107-Gal4/UAS-mCD8-GFP/UAS-human PrP-N159D-D167S-attP2*) or 3x (*OK107-Gal4/UAS-mCD8-GFP/UAS-human PrP-N159D-D167S-N174S-attP2*) show small projections at day 1 but slower degeneration. (M) Quantification of axonal projections. Statistical significance between groups is shown by the connecting letters. Levels not connected by the same letter are significantly different. *P*-value for different letter groups is <0.0001, except for B and C (*P*=0.0037), and between CD and D (*P*=0.021). (N) Area differential day 40 – day 1 for each condition. Only control flies and flies expressing the 3x mutant show an expansion of axonal projections over time. Data from 10-12 brains were analyzed by two-way ANOVA and adjusted for multiple comparisons.

We last examined the consequence of expressing the new human PrP constructs in the mushroom bodies. Fig. 8 shows the axonal projections of the mushroom body neurons, which split into dorsal (α) and medial (β and γ) lobes. We measured the surface of the projections in each genotype in young (day 1 post eclosion) and old (day 40) flies. Control 1-day-old control flies showed robust axonal projections (Fig. 8A) that expand in surface in 40-day-old flies (Fig. 8G,M,N) (Sanchez-Garcia and Fernandez-Funez, 2018; Fernandez-Funez et al., 2010). One-day-old flies expressing PrP-V129 exhibited thinner axonal projections (Fig. 8B,M). By day 40, these flies showed extensive degeneration: loss of α lobes and widespread membrane blebbing (Fig. 8H,M). One-day-old flies expressing human PrP N159D, D167S, 2x or 3x mutants exhibited similar axonal projections compared to young flies expressing PrP-V129 (Fig. 8C-F,M). By day 40, all the mutants showed extensive blebbing, but the preservation of the lobes was different. The area covered by axonal projections of 40-day-old flies expressing PrP-N159D was similar to that in controls expressing PrP-V129 (Fig. 8I,M,N), flies expressing D167S or 2x exhibited significantly larger lobes (Fig. 8J,M,N). Flies expressing the 3x mutant showed expansion of the mushroom body lobes as they age (Fig. 8L,M,N), but they were still smaller than in controls. Details for the statistical analysis are shown in Table S6. Overall, the analysis of mushroom body degeneration showed that human PrP is highly toxic to brain neurons starting during development and continuing with extensive degeneration during aging, but constructs carrying the D167S substitution showed moderate protection.

## DISCUSSION

Here we described the characterization of new genetic tools in *Drosophila* with the potential to dissect the mechanism underlying PrP toxicity. We generated codon-optimized rodent and human PrP constructs and integrated them in the same attP2 landing site. Since two copies of rodent PrP induced no eye toxicity, it is unlikely that higher expression levels alone are responsible for the new phenotypes of human PrP. Instead, it is likely that human PrP acquires conformations responsible for their high toxicity in flies. Differences in the biogenesis of rodent and human PrP are evidenced by different glycosylation patterns and subcellular distributions. PrP glycosylation isoforms indicate different exposure of the glycosylation sites. Retention of rodent PrP in the secretory pathway indicates slow or inefficient maturation resulting in reduced membrane expression. Previous studies have shown that no immature glycosylated hamster PrP is detected and PrP is present in lipid rafts (Fernandez-Funez et al., 2010, 2009), suggesting that rodent PrP can complete its maturation and secretion. The partial retention of the secretory pathway may result in some degradation, explaining the higher protein levels of human PrP despite identical levels of mRNA. In addition to the eye phenotype, human PrP induced other novel phenotypes: lethality, aggressive locomotor dysfunction and elimination of the mushroom bodies. Importantly, human PrP was sensitive to mild proteinase K digestion, indicating no accumulation of spontaneous PrP^res^. Flies were not expected to generate prions spontaneously, just like WT or transgenic mice do not develop prions spontaneously. Transmissible prions require specific structural properties that can be replicated from seeds but are rarely produced *de novo* in humans and some ungulates, and require extensive incubation. Other labs have reported the ability of flies to replicate mammalian prion seeds in transmission experiments, demonstrating a good cellular environment for PrP conversion (Thackray et al., 2014, 2012a). The lack of spontaneous PrP^res^ is consistent with the idea that neurotoxicity is caused by different conformations of transmissible PrP (Sandberg et al., 2014, 2011). The lack of spontaneous PrP^res^ in flies suggests that responsible work can be done with these flies at enhanced Animal Biosafety Level 2 (ABSL2).

As a proof-of-concept for the sensitivity of these flies to extrinsic factors modulating PrP toxicity, we examined the functional interaction of human PrP with Aβ42 and the UPR. Laurin and colleagues have found that Aβ42 binds the unstructured N-terminal domain of PrP, a novel interaction proposed to mediate Aβ42-dependent inhibition of long-term potentiation (Laurén et al., 2009). Despite initial resistance (Calella et al., 2010; Kessels et al., 2010; Balducci et al., 2010), this interaction was confirmed by using different techniques, although studies still disagree on the functional meaning of this interaction (Chen et al., 2010; Zou et al., 2011; Gimbel et al., 2010; Gunther and Strittmatter, 2010; Balducci et al., 2010). The native PrP conformation is proposed to work as a scaffold that brings together membrane proteins in lipid rafts, including glutamate and lamin receptors (Zhang et al., 2019). The Aβ42 – PrP interaction stimulates glutamate receptors, whereas the interaction with lamin receptors internalizes the complexes, resulting in significant ER stress due to retrograde transport of Aβ42 (Casas-Tinto et al., 2011). Here, we show that human PrP, but not hamster or mouse PrP, increased Aβ42 toxicity. Interestingly, a similar functional interaction was described recently in the *Drosophila* brain between Aβ42 and ovine PrP (REF) (Younan et al., 2018). This is consistent with our finding that human PrP has more binding sites for Aβ42 (six) than mouse PrP (one) (Zou et al., 2011). Additionally, Aβ42 and human PrP induced similar, although not identical, eye phenotypes in flies suggesting that Aβ42 and PrP perturb similar gene networks in the eye, including ER stress.

Our analysis of the UPR showed that silencing Ire1α or XBP1 robustly enhances PrP toxicity, indicating the protective activity of this pathway. Surprisingly, overexpression of XBP1 had no effect on PrP toxicity. XBP1 was expected to show protective activity because its downstream targets support ER proteostasis. In our previous work we have shown that XBP1s overexpression is protective in flies expressing Aβ42 (Casas-Tinto et al., 2011). XBP1 also shows protective activity against other stressors in *C. elegans* (Taylor and Dillin, 2013). A general assumption is that all UPR branches are equally responsive and protective against all triggers. We show here that human PrP activated the Ire1α branch in flies, yet Aβ42 induced a stronger response. This is consistent with our previous findings that cultured cells exposed to oligomeric amyloids, with Aβ42 and α-synuclein induce stronger activation of Ire1α than the PrP106-126 fragment and the British amyloid peptide (Castillo-Carranza et al., 2012). Thus, human PrP might only be a moderate inducer of the Ire1α branch while robustly inducing the PERK branch. Notably, this robust PERK activation shut down translation through phospho-eIF2α, thus preventing the transcriptional response of XBP1.

Our main finding is that silencing of either PERK or of its effector ATF4 robustly suppressed human PrP toxicity. This robust protective activity is consistent with recent findings in prion-infected mice (Hughes and Mallucci, 2019; Moreno et al., 2012). We report here for the first time a similar protective activity of ATF4, indicating that modulation of ATF4 activity elicits a full protective activity equivalent to silencing the upstream sensor. Moreover, increased PERK or ATF4 activity perturbed the eye, but only ATF4 showed a strong genetic interaction with PrP since PERK was able to disrupt eye development on its own. The robust ATF4 interactions with PrP were surprising since the PERK maladaptive activity is proposed to emanate from the phospho-eIF2α, a direct PERK target. It is prudent to remember that the PERK pathway is slightly different in flies and mammals. Flies do not express a CHOP orthologue, which is an ATF4 target with deleterious activities, eliminating CHOP as the effector of ATF4 toxicity in flies. Additionally, PPP1R15 in flies is activated directly by PERK and through the same translational mechanism as ATF4 (Kang et al., 2015). We show here that 4E-BP, an ATF4 target discovered in the General control non-repressible 2 (Gcn2) nutrition-sensing pathway (Kim et al., 2020; Malzer et al., 2018; Kang et al., 2017; Vasudevan et al., 2017), was also involved in PrP toxicity. eIF2α is a key regulator of translation, which is activated by PERK, Gcn2 and two additional kinases, and eIF2α downstream effectors are likely shared by the stress pathways. Thus, the ATF4 transcriptional target 4E-BP is activated in flies that express human PrP, resulting in chronic block of translation by binding to eIF4E. It is likely that the sequential activity of phospho-eIF2α and 4E-BP produces a robust translational inhibition to ensure recovery from ER stress or nutritional deficiency. Silencing of 4E-BP suppresses PrP toxicity by allowing translation to proceed, but this is expected to have no impact on the levels of phospho-eIF2α, which can still block translation. Since phospho-eIF2α can be rapidly dephosphorylated by PPP1R15, removing 4E-BP can achieve robust suppression of PrP toxicity on its own despite being downstream of phospho-eIF2α. We will further investigate the interplay between PERK, Gcn2, ATF4, eIF2α and 4E-BP in follow-up studies.

We do not yet fully understand the exact intrinsic mechanisms mediating the conformational dynamics of PrP and how they translate into different toxicity, disease susceptibility or strain variability. While a few amino acid differences between mammalian PrPs are responsible for conformational differences, it remains challenging to pinpoint how specific amino acids contribute to PrP conformation (Myers et al., 2020). The new *Drosophila* models enable mechanistic studies into sequence-structure-phenotype analyses through the efficient introduction of candidate mutations into the human PrP backbone. In a previous report we have shown that two humanized mutants, i.e. dog PrP-D159N and horse PrP-S167D, turned these non-toxic PrPs into toxic ones (Sanchez-Garcia and Fernandez-Funez, 2018). We have predicted that the corresponding protective residues from dog and horse PrP into human PrP are protective. D167S is mildly protective in the eye and the mushroom bodies; yet N159D only shows weak protection of mushroom body neurons. Interestingly, the combinations N159D-D167S or N159D-D167S-N174S showed similar protective activity as D167S alone. These results provide valuable lessons regarding the rules that govern PrP misfolding and toxicity. First, single amino acid changes are not enough to alter the high structural dynamics of human PrP. Second, N159D and D167S are not known to form distinct secondary or tertiary structures in dog and horse PrP (Pérez et al., 2010; Lysek et al., 2005), suggesting that they do not introduce significant changes in human PrP. In contrast, S174 participates in a helix-capping domain that stabilizes helix 2 in rabbit PrP (Khan et al., 2010). However, addition of N174N to the 3x mutant had a small effect. Third, combining amino acid changes from different animals did not increase the conformational stability of human PrP. A more likely strategy would combine conservative changes from the same animal to recreate local structural features from dog, horse or rabbit PrP. We are currently testing several such combinations, including Y225A from rabbit (R.M.M., Aliciarose John, Weiguanliu Zhang, Wen-Quan Zou, Alessandro Cembran and P.F.F., unpublished observations) and others. The ability to efficiently test candidate mutations *in vivo* will eventually provide answers to the questions posed above, i.e. regarding the correlations between genotype, morphotype and phenotype.

## MATERIALS AND METHODS

### Sequence alignment and 3D protein visualization

The alignments of the globular domain of human, hamster, mouse, dog, horse and rabbit prion protein sequences were done using ClustalW2 (www.ebi.ac.uk/Tools/clustalw2). We used human PrP as reference, and amino acid numbering for all species refers to the corresponding amino acid in human PrP (see Fig. 1A). PrP amino acid sequences were obtained from NCBI with the following accession numbers: AAH22532 (human), AAA37092 (Syrian hamster), and AAA39996 (mouse), AAD01554 (rabbit), ACG59277 (horse), and ACO71291 (dog). The color-coded amino acids in Fig. 1A indicate properties relevant for protein structure (size and charge). To generate 3D views of human, mouse and Syrian hamster PrP, we opened in PyMOL (pymol.org) the published NMR structures for human (1QM2), mouse (1XYX), hamster (1B10), rabbit (2FJ3), horse (2KU4) and dog (1XYK) PrP deposited in the RSCB Protein Data Bank (rcsb.org). We displayed the PrP globular domain showing only relevant amino acids to optimize their visualization and the β2-α2 loop using the Surface and Mesh views.

### Generation of transgenic flies and genetics

#### Random insertions

Flies carrying the human PrP-WT (V129) construct in a random insertion were described previously (Fernandez-Funez et al., 2017). We generated flies carrying human PrP-M129 in a random insertion following the same procedures described above.

#### attP2 insertions

Constructs carrying human PrP-M129, human PrP-V129, hamster PrP-WT and mouse PrP-WT, as well as the human PrP mutants PrP-N159D, PrP-D167S, PrP-N159D-D167S (double) and PrP-N159D-D167S-N174S (triple, 3x) (all in V129 background) were chemically synthesized by Integrated DNA Technologies (IDT) using codon-optimized sequences for *Drosophila*. Assembled sequences were cloned between *XhoI* and *Xba*I sites onto the pJFRC7-20XUAS-IVS-mCD8:GFP *Drosophila* expression vector (Pfeiffer et al., 2010; Addgene #26220) after removing the mCD8:GFP transgene. The final constructs were sequenced to verify their integrity. The pUAST-based constructs were injected into *yw* embryos at Rainbow Transgenics following standard procedures (Rubin and Spradling, 1982) to generate multiple independent transgenic lines for each plasmid. Two independent strains were generated for each construct since they are all inserted in the same *attP* locus.

The driver strains *GMR-Gal4* (retina, all eye cells) (Mathew Freeman, University of Oxford), *OK107-Gal4* (mushroom bodies) (Connolly et al., 1996), *Elav-Gal4* (pan-neural) (Lin and Goodman, 1994), *Elav-GS* (pan-neural, GeneSwitch) (Roman et al., 2001), the reporters *UAS-LacZ UAS-mCD8-GFP*, *UAS-Rab4-RFP*, *Rab11-GFP*, *Sec16-Tomato* and *γCOPII-GFP*; the TRiP RNAi lines *Ire1*α*^HMC05163^*, *XBP1^JF02012^*, *PEK^HMJ02063^*, *crc/ATF4^JF02007^*, *eIF2*α*^GLC01598^*, *PPP1R15^HMS00811^* and *Thor^HMS06007^* (*4E-BP*); and *UAS-PERK* (*pek*), *UAS-ATF4* and *UAS-Thor* were obtained from the Bloomington *Drosophila* Stock Center (fly.bio.indiana.edu). RNAi alleles for *PERK*, *ATF4*, *PPP1R15* and *eIF2*α were obtained from the Vienna *Drosophila* Stock Center (stockcenter.vdrc.at/control/main) (see Table S4). Transgenic flies expressing human Aβ42 and UAS-mouse XBP1s were described previously (Casas-Tinto et al., 2011) and the *XBP-GFP* sensor was obtained from HD Ryoo (Ryoo et al., 2007). UAS alleles for *ATF4*, *eIF2*α and *PPP1R15* were obtained from FlyORF (flyorf.ch/index.php). Fly stocks were maintained on standard *Drosophila* medium at 25°C. For experiments, homozygous females for the *Gal4* strains were crossed with *UAS* males to generate progeny expressing *PrP* in the desired tissue. Crosses were placed at 25°C for 2 days and transferred to 27°C until the progeny completed development; adult flies were aged at 27°C, unless otherwise indicated.

### Characterization of eyes

We expressed all constructs in the eye under the control of *GMR-Gal4*. Crosses were performed at 25°C for 2 days and progeny was raised at 28°C; adult flies were collected at day 1. Images were collected from flies with representative phenotypes out of large progenies of more than 10 females. To image fresh eyes, we froze the flies at −20°C for at least 24 h and collected images as *Z*-stacks with a Leica Z16 APO using a 2× Plan-Apo objective. Flattened in-focus images were produced with the Montage Multifocus module of the Leica Application Software. Fresh eyes were scored for changes with respect controls: N, no change; E, enhancer; S, suppressor. Changes in the eyes were also scored in four categories from 0 to 3 in each: eye size, organization, pigmentation and lethality, with 0=no change and 3=maximum change. Changes were assessed from large progenies (at least ten flies) and scores reflect representative and highly reproducible changes. For scanning electron microscopy (SEM), flies were serially dehydrated in ethanol, critical point dried and metal-coated for observation in a Jeol JSM-6490LV. For transmission electron microcopy, we collected flies of the appropriate genotype 1-day post eclosion, fixed the heads in 3% glutaraldehyde overnight, washed in phosphate buffer, post-fixed in 1% OsO_4_, dehydrated in ethanol and propylene oxide, embedded in resin and, subsequently, mounted the heads in molds as described previously (Fernandez-Funez et al., 2000). Blocks were then cut into semithin sections (1 μm), stained with toluidine blue and imaged in a Nikon Eclipse Ni microscope with a 100× Plan Apo oil 1.4 NA objective. For ultrastructural analysis of the eyes, we collected ultra-thin sections (70 nm), stained them, and imaged the samples at magnifications between 2500× and 25,000×, using a Jeol JEM-1400PLUS TEM at the University Imaging Centers.

### *Drosophila* homogenates and western blotting

Ten flies per genotype and time point were used for analysis. Fly heads were homogenized in 30 µl of RIPA buffer containing complete protease inhibitors (Roche) with a motorized pestle and centrifuged for 1 min at 1000 rpm. 25 µl of supernatant was mixed with loading buffer, resolved by SDS-PAGE on 4-12% Bis–Tris gels (Invitrogen) under reducing conditions and electro-blotted onto nitrocellulose membranes. Membranes were blocked in TBS-T containing 5% non-fat milk and probed with the following primary antibodies: anti-PrP clone 8H4 (1:10,000, Millipore, batch 099M4844V), anti-PrP clone 3F4 (1:10,000, Millipore, Lot 3150381), anti-β-Tubulin (1:50,000, Invitrogen, clone 2 28 33). The secondary antibody used was anti-mouse-HRP (1:4000) (Jackson ImmunoResearch, Lot 138817). Antibodies were validated by using control lanes (non-PrP) and verification of expected profile. Immunoreactive bands were visualized by enhanced chemiluminescence (ProSignal Dura ECL, Genesee). The protein biochemistry protocols have been described in more detail by Sanchez-Garcia et al. (2013). For protease-resistance assays, fly brain homogenates were incubated with proteinase K at concentrations between 0 and 15 µg/ml for 30 min at 25°C. Digestions were stopped by adding 2 mM PMSF and analyzed by western blotting using the anti PrP antibody 3F4. To quantify signal intensities, films were scanned at high resolution, band intensities measured and normalized against background and internal control, graphed in Excel and analyzed using two sample *t*-test.

### Quantitative RT-PCR (qPCR)

Ten male flies 1-2 days post eclosion were used per genotype for analysis. Fly heads were homogenized in 100 µl RTL buffer from RNeasy kit (Qiagen) using a motorized pestle. An additional 250 µl RTL buffer were added and then centrifuged for 3 min at 21,000 ***g***. Supernatant was collected, placed in a new tube, and used for RNA extraction using the RNeasy kit. Additional DNase (DNase I, NEB) treatment and ethanol precipitation was performed. Omniscript Reverse Transcription Kit (Qiagen) was used for cDNA synthesis following the manufacturer’s protocol and using 50 ng RNA for each sample. cDNA was then diluted 5× before qPCR.

qPCR was performed on a Roche Lightcycler 480 Instrument II and using SYBR Green I Master Mix (Roche), following the manufacturer’s protocol. PrP primers were designed to amplify the same sequence. The housekeeping *Drosophila* gene *Glyceraldehyde 3-phosphate dehydrogenase* (*GAPDH*) was used as an internal control. Negative RT controls were run to eliminate contaminating genomic DNA. The following primers were used: human PrP forward 5′-GCGGCAATCGTTACCCTCCTC-3′; human PrP reverse 5′-ACTGGGCTTATTCCACTGGGAGT-3′; mouse PrP forward 5′-GTAACCGCTACCCACCGCAAG-3′; mouse PrP reverse 5′-TGGTTTGCTGGGCTTGTTCCA-3′; hamster PrP forward 5′-TCCCCAGGAGGTAATCGGTATCCT-3′; hamster PrP reverse 5′-TGGTTATGAGTGCCTCCACCCT-3′; GAPDH forward 5′-TAAATTCGACTCGACTCACGGT-3′; GAPDH reverse 5′-CTCCACCACATACTCGGCTC-3′. Each genotype was examined in three biological replicates together with three technical replicates for each. The −ΔΔct method was used for data analysis and represented as the relative expression to human PrP.

### Immunofluorescence, microscopy, image display and analysis

Whole-mount immunohistochemistry of fixed larval brains or eye imaginal discs was conducted by fixing in 4% formaldehyde, washing with PBT, and blocking with 3% bovine serum albumin before incubating with the primary antibody as described previously (Fernandez-Funez et al., 2010). We incubated first with the 8H4 anti-PrP antibody (1:2000 dilution) followed by the secondary antibody anti-mouse-Cy3 (Molecular Probes) at 1:1000 dilution. We mounted the stained tissues in Vectashield antifade (Vector) mounting medium for microscopic observation and documentation. We collected fluorescent images in an LSM 710 Zeiss confocal system using 10× (NA 0.45; air), 20× (NA: 1.0; air) and 63× (NA 1.4; oil) objectives in thick samples as *Z*-stacks. All genotypes for the same experiment were imaged with the same settings. From the *Z*-stacks, we created maximum intensity projections or extracted single planes images using the Zeiss Zen software. These images were combined into figures using Adobe Photoshop; processing included trimming of non-informative edges and brightness/contrast adjustment to whole images. The cartoon for the UPR pathway was created in Adobe Illustrator. Whole-mount adults brains labeled with mCD8-GFP were imaged at day 1 post eclosion with the 10× objective.

#### Subcellular localization

We co-expressed the *PrP* constructs along with *mCD8-GFP*, *UAS-Rab4-RFP*, *Rab11-GFP*, *Sec16-Tomato* and *γCOPII-GFP* in interneurons of the larval ventral ganglion under the control of *OK107-Gal4* (*UAS-reporter-GFP; OK107-Gal4/UAS-PrP*). Regions containing interneurons were imaged with a 63× objective and 1.5× digital zoom. Images displayed in the figure are representative single planes extracted from the stacks. For the analysis of overlap, we created the maximum intensity projections, obtained the signal intensity for 20-30 individual cells before and after subtracting the signal for both channels, normalized the signal for the surface (neuron size) and calculated the fraction of overlap to total. Differences between rodent and human PrP were calculated by *t*-test. For CD8-GFP-GFP, mouse and hamster PrP were statistically comparable and were aggregated to compared to both human PrPs.

#### XBP-GFP

Eye imaginal discs expressing XBP-GFP in the eye under the control of GMR-Gal4 were combined with LacZ, Aβ42 or PrP. Imaginal discs were imaged with the 20X objective. Signal intensity for flattened images was extracted in Adobe Photoshop 2021 following manual outlining of the anterior region of the eye disc. One-way ANOVA analysis was conducted in JMP Pro 16. Following the finding that the averages were statistically significant, we performed a Tukey-Kramer post hoc pair-wise analysis of significance to determine which pairs were statistically different while reducing the false positive due to the analysis of multiple pairs. To simplify the multiple group comparisons, we displayed the connecting letters report, i.e. groups with different letters correspond to statistically significant differences, with the differences being proportional to the distance between the letters. See details in Table S3.

#### Mushroom body degeneration

We crossed *OK107-Gal4; mCD8-GFP* flies with *LacZ* alone (negative control) or with *PrP* constructs (*UAS-mCD8-GFP; OK107-Gal4/UAS-PrP*) at 27°C. Adult flies were collected at days 1 and 40 post eclosion and imaged with the 63× objective. The surface for mushroom body axonal projections was manually outlined and measured in Photoshop from 15-20 mushroom bodies. Image analysis data were exported to Excel to calculate averages, standard deviations and create graphs. Two-way ANOVA analysis of the effects of genotype and age was conducted using JMP Pro 16. ANOVA showed significant effects of genotype (*F*_5, 121_=106.79, *P*<0.001), age (*F*_1, 121_=5.32, *P*<0.05) and the interaction of genotype with age (*F*_5, 121_=48.35, *P*<0.001). Following ANOVA, *post hoc* pairwise *t*-test analyses were conducted in JMP Pro 16. *t*-tests were corrected using Holm's method (Sokal and Rohlf, 2012).

### Behavior, locomotor assays

For the strong random human PrP insertion, we performed locomotor assays following conditional expression in adult flies by using the Elav-GS system. For this, we combined *Elav-GS* with *UAS-LacZ*, *UAS-hamster PrP-random* and *UAS-human PrP-random*, and placed the crosses in fly medium without the activator RU486 (Sigma). When the adult flies eclosed, we collected 20 females per replicate and split them in two groups, i.e. one in vials without RU486 and one with RU486. Then, we examined the ability to move vertically in an empty vial (climbing assay) at 28°C (Le Bourg and Lints, 1992). Briefly, 20 newborn adult females were placed in empty vials in duplicates and forced to the bottom by firmly tapping against the surface. After 10 s, the number of flies climbing higher than 5 cm was recorded. This was repeated eight times to obtain the average climbing index per day. At the end of the assay, the climbing index [(flies above line:total flies)×100] was plotted as a function of age in Excel.

Climbing index data were fitted to either a 3-parameter logistic (LacZ±RU HaPrP±RU, and HuPrP−RU) or 3-parameter first-order decay kinetics model (HuPrP+RU) using JMP Pro 16 (SAS Institute). Fitted curves were used to predict the time in days to climbing index values (age-specific climbing index) of 90, 75, 50, 25 and 10 (α=0.05) (Table S1). Prediction using increasingly more stringent α values did not change the prediction. Prediction formulas and parameters for each genotype-RU combination are listed in Table S2. We tested the null hypothesis that RU had no effect on age-dependent climbing ability using a single sample *t*-test. Single-sample *t*-test with 5 degrees of freedom (critical value=2.015 at α=0.05) showed a significant negative effect of RU on age-dependent climbing effect when combined with HuPrP. We calculated t-scores instead of z-scores because sample size was ≤30. Additionally, we computed the area under each climbing index curve. Single-sample *t*-test again confirmed a significant negative effect of the HuPrP+RU combination on the climbing index.

## Supplementary Material

Supplementary information
