# Supplementary material for: New Drosophila models to uncover the intrinsic and extrinsic factors that mediate the toxicity of the human prion protein
Source: Dis Model Mech. 2022 May 4;15(4):dmm049184. doi: 10.1242/dmm.049184 (PMC9093039; doi:10.1242/dmm.049184)
Supplement: Supplementary information [file dmm-15-049184-s1.pdf]

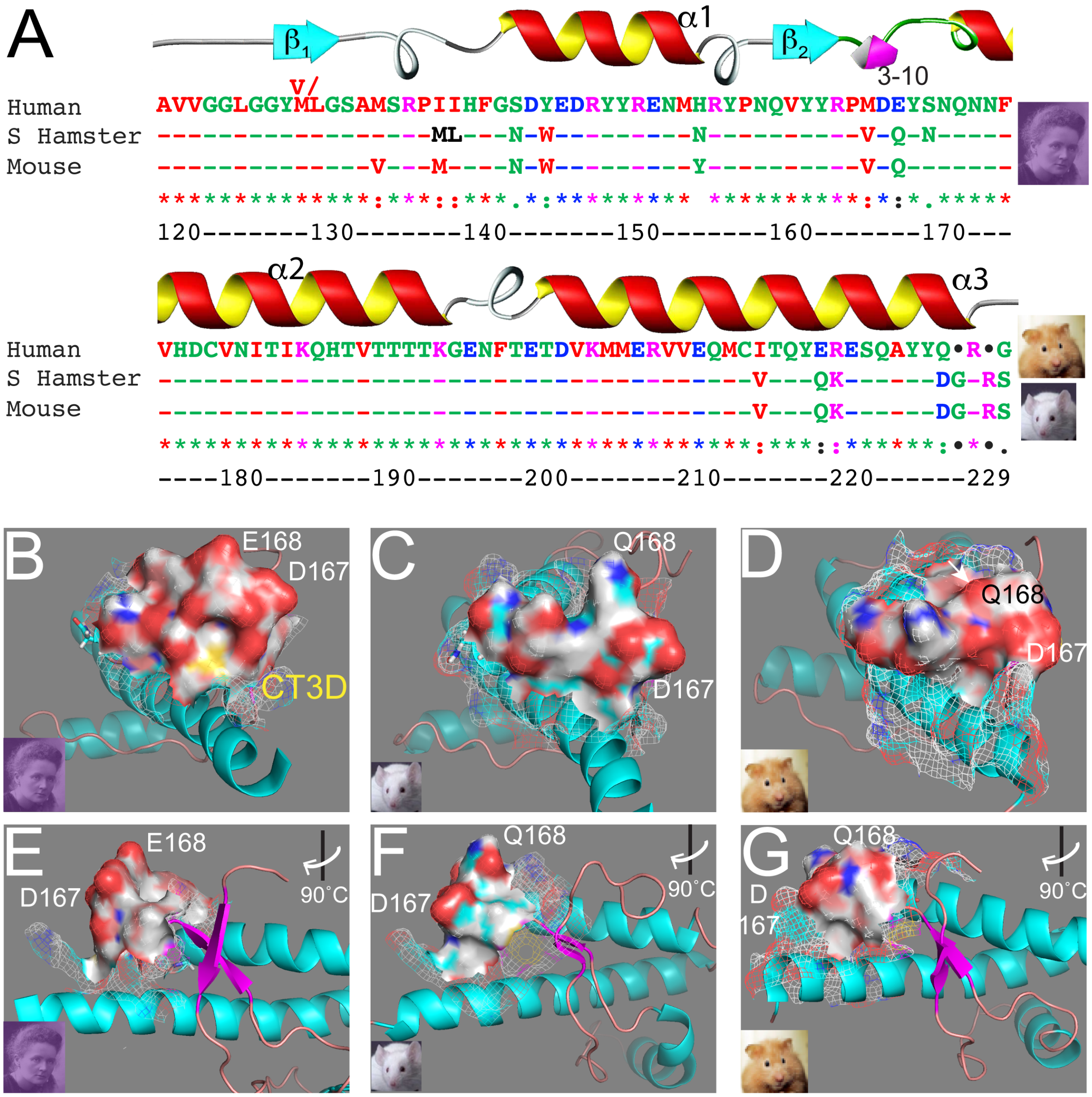

Fig. S1. Sequence / structure differences between human and animal PrP. A, Sequence alignment of the C-terminal globular domain of PrP from human, Syrian hamster, and mouse. Amino acid numbering corresponds to human PrP throughout to avoid confusion. The alignment shows high overall conservation with most variation clustered in the β2-α2 loop and distal helix 3. **B-G**, Surface and Mesh views for the β2-α2 loop, front view (B-D) and side view (E-G). In human PrP, the loop is vertical and tall, with two acidic residues sticking upwards (B and E). In mouse PrP, the loop is not as tall, with D167 shifted to a lower position (C and F). In hamster PrP, the loop is flat and closer to helix 3 (D and G).

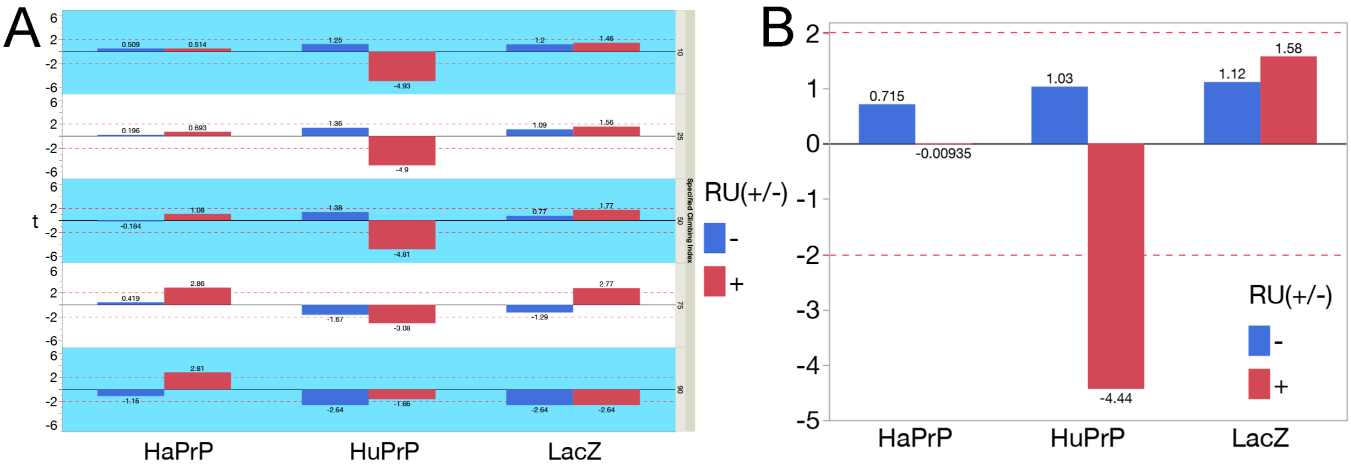

Fig. S2. Statistical analysis of locomotor activity. A, Single-sample t-test analysis of age-dependent climbing index. HuPrP with RU has a significant negative effect on age-specific climbing index. Bars represent t-score. **B**, Area under the curve. Dotted red-line represents the critical value of 2.015 for t-test with 5 degrees of freedom ($p<0.05$). Single-sample t-test analysis of area under curve for the climbing index. HuPrP with RU has a significant negative effect on area under the climbing index curve. Bars represent t-score. Dotted red-line represents the critical value of 2.015 for t-test with 5 degrees of freedom

Disease Models & Mechanisms • Supplementary information

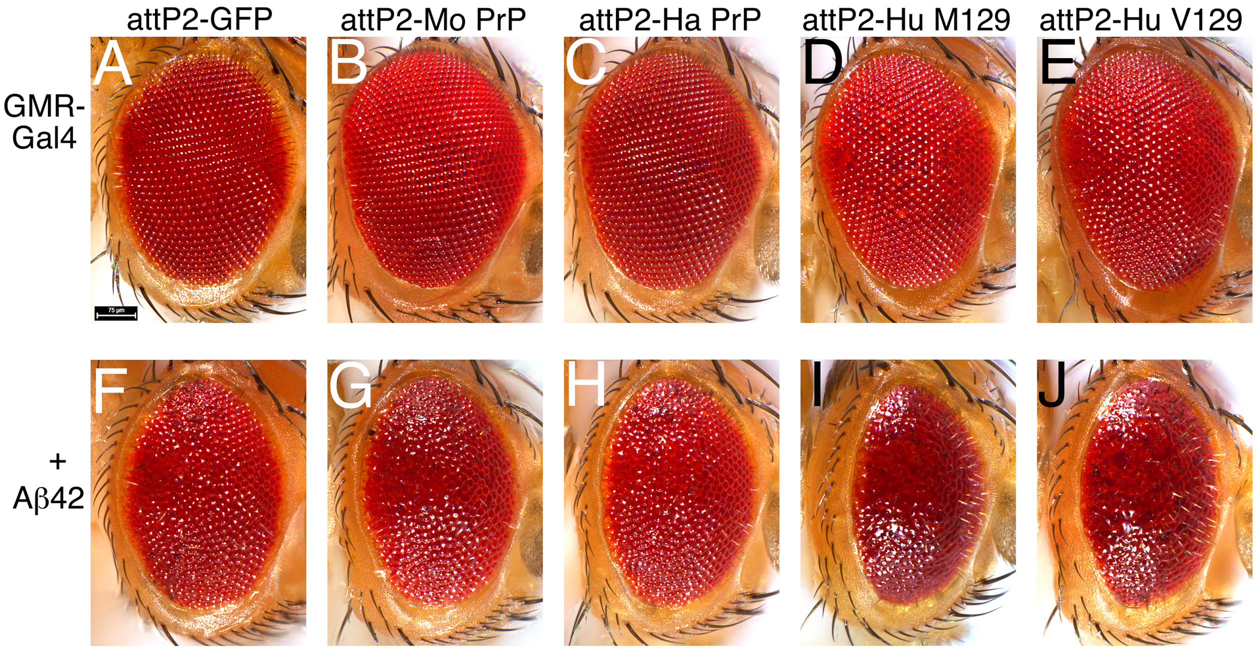

Fig. S3. Human PrP enhances the toxicity of Aβ42. A-J, Micrographs of fresh eyes expressing mCD8-GFP, hamster PrP, mouse PrP, human PrP-M129, or human PrP-V129 alone (A-E) or in combination with Aβ42 (F-J) in the eye under the control of *GMR-Gal4* at 25˚C. **A**, Control eyes from flies expressing mCD8-GFP (*GMR-Gal4 / UAS-mCD8-GFP-attP2*). **B and C**, Eyes from flies expressing mouse or hamster PrP (*GMR-Gal4 / UAS-mouse PrP-attP2 and GMR-Gal4 / UAS-hamster PrP-attP2*) are normal. **D and E**, Eyes from flies expressing human PrP (*GMR-Gal4 / UAS-human PrP-M129-attP2 and GMR-Gal4 / UAS-human PrP-V129-attP2*) show mild disorganization. These phenotypes are weak because the expression of PrP constructs is lower at 25˚C. **F**, The eyes from flies co-expressing GFP and Aβ42 (*GMR-Gal4 / UAS-mCD8-GFP-attP2/ UAS-Aβ42*) are disorganized and have necrotic spots. **G and H**, The eyes from flies co-expressing rodent PrP with Aβ42 (*GMR-Gal4 / UAS-mouse PrP-attP2 / UAS-Aβ42 and GMR-Gal4 / UAS-hamster PrP-attP2 / UAS-Aβ42*) are similar to those in F. **I and J**, The eyes from flies co-expressing human PrP and Aβ42 (*GMR-Gal4 / UAS-human PrP-M129-attP2 / UAS-Aβ42 and GMR-Gal4 / UAS-human PrP-V129-attP2 / UAS-Aβ42*) are smaller and highly disorganized (glassy).

Disease Models & Mechanisms • Supplementary information

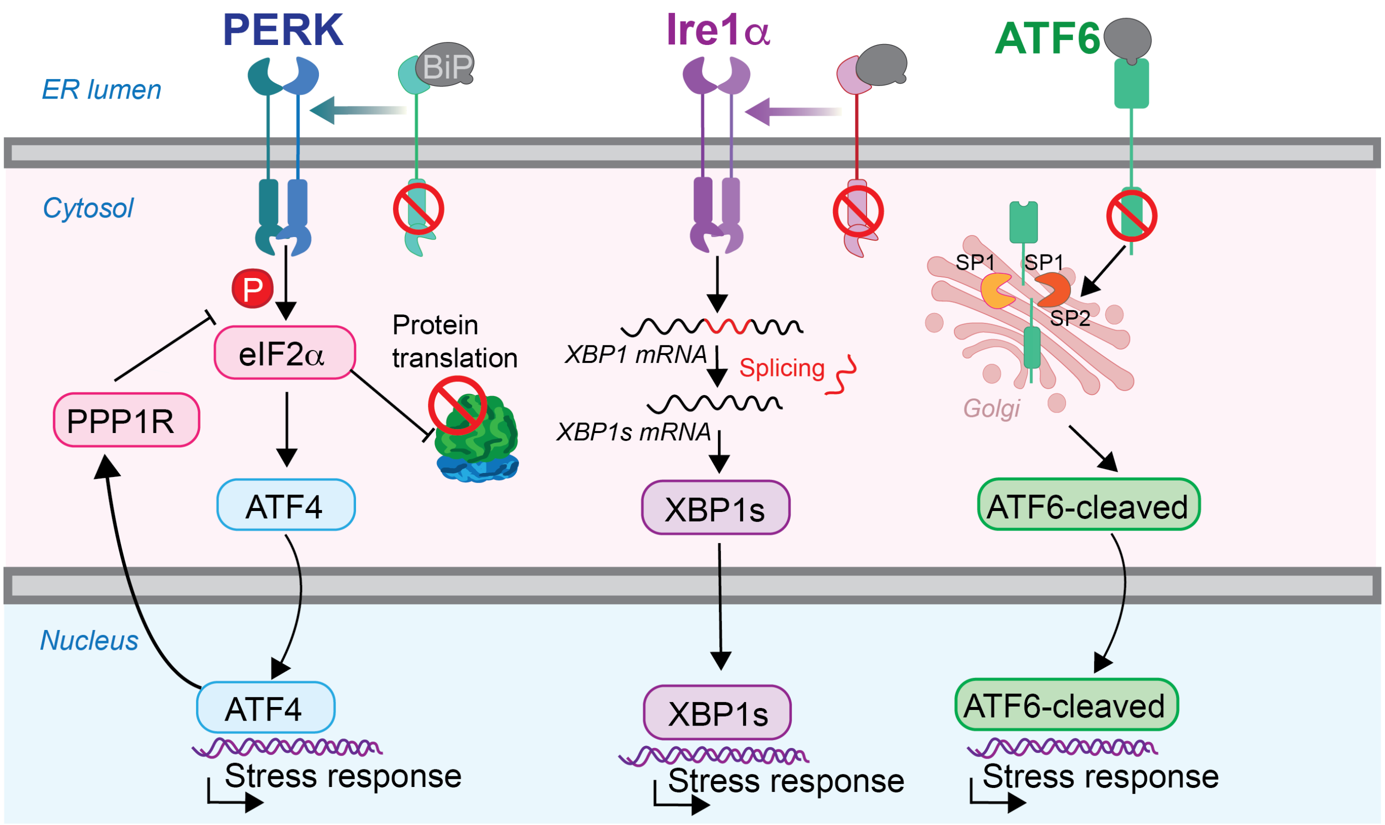

Fig. S4. Summary of the three UPR branches. PERK, Ire1 and ATF6 are three sensors of ER stress that are activated when the chaperone BiP releases the sensors due to an increase of misfolded proteins in the ER. Each sensor activates a different branch of the UPR consisting of different mechanisms of activation and downstream effectors. The overall consequence is acutely blocking translation and increasing the ER biosynthetic and folding capacities.

Disease Models & Mechanisms • Supplementary information

Table S1. Predicted age at specified climbing indices (α = 0.05).

Genotype	RU(+/-)	Climbing Index	Predicted Age (Days)	Std Error	Lower 0.95	Upper 0.95
HaPrP	-	90	1.09	1.27	-1.58	3.76
HaPrP	-	75	6.38	0.38	5.58	7.18
HaPrP	-	50	11.86	0.35	11.12	12.61
HaPrP	-	25	17.42	0.48	16.41	18.42
HaPrP	-	10	22.99	0.90	21.10	24.88
HaPrP	+	90	4.00	1.84	0.12	7.88
HaPrP	+	75	10.23	0.55	9.07	11.39
HaPrP	+	50	14.76	0.40	13.91	15.62
HaPrP	+	25	18.94	0.52	17.83	20.05
HaPrP	+	10	23.01	0.90	21.12	24.90
HuPrP	-	90	--	--	--	--
HuPrP	-	75	3.09	3.20	-3.66	9.85
HuPrP	-	50	15.47	0.44	14.54	16.40
HuPrP	-	25	20.99	0.43	20.07	21.90
HuPrP	-	10	25.77	0.78	24.13	27.41
HuPrP	+	90	0.72	0.03	0.64	0.79
HuPrP	+	75	0.87	0.03	0.81	0.93
HuPrP	+	50	1.21	0.02	1.16	1.26
HuPrP	+	25	1.80	0.05	1.69	1.91
HuPrP	+	10	2.56	0.10	2.35	2.77
LacZ	-	90	--	--	--	--
LacZ	-	75	3.68	1.13	1.30	6.07
LacZ	-	50	14.06	0.34	13.35	14.77
LacZ	-	25	20.16	0.35	19.43	20.90
LacZ	-	10	25.60	0.64	24.24	26.95
LacZ	+	90	--	--	--	--
LacZ	+	75	10.07	0.63	8.74	11.41
LacZ	+	50	16.36	0.43	15.44	17.27
LacZ	+	25	21.59	0.53	20.48	22.71
LacZ	+	10	26.56	0.94	24.58	28.53

Disease Models & Mechanisms • Supplementary information

Table S2. Climbing Index prediction formulas and parameters.

Prediction Model	Prediction formula	Genotype	RU(+/-)	θ1	q2	q3
3-parameter logistic	*Log((q1/ Climbing Index)/ q2)/ q3*	HaPrP	-	101.05	0.1	0.2
		HaPrP	+	94.19	0.02	0.27
		HuPrP	-	76.96	0.01	0.24
		LacZ	-	79.92	0.03	0.21
		LacZ	+	89.09	0.02	0.23
3-parameter 1st order decay kinetic	*Log((Climbing Index - q3)/ q1)/ (-q2)*	HuPrP	+	209.74	1.18	-0.3

Table S3. ANOVA analysis for XBP-GFP expression.

Comparisons for all pairs using Tukey-Kramer HSD

Connecting Letters Report

Level				Mean (Mean signal)	Mean (Integrated density)
Abeta42	A			12.749299	1298165.5
PrP-V129		B		10.634235	966270.2
Control			C	6.193575	540884.8

Levels not connected by same letter are significantly different.

Ordered Differences Report (mean signal)

Level	- Level	p-Value (mean signal)	p-Value (Integrated density)
Abeta42	Control	<.0001	<.0001
PrP-V129	Control	<.0001	0.0005
Abeta42	PrP-V129	0.0004	0.0008

Table S4. Summary of interactions with the UPR-PERK components

27˚C Stock#	**UAS-LacZ** 3955	**GD-Ire1αi** 39561	**TRiP-Ire1αi** 62156	**KK-XBP1i** 109312	**TRiP-XBP1i** 25990	**UAS-XBP1** 60730	**UAS-mXBP1s** Casas-Tinto 2011	**KK-PERKi** 110278	**TRiP-PERKi** 42499	**UAS-PERK** 76248
**GMR**	N0000	N0000	N0000	N0000	N0000	N0000	N0000	N0000	N0000	E3332
**GMR PrP**	E23000	E3311	E2201	E3300	E2300	N0000	N0000	S3300	S3300	E3332
	**KK-ATF4i** 109014	**TRiP-ATF4i** 25985	**UAS-ATF4** FlyORF	**UAS-ATF4** 81650	**TRiP-eIF2αi** 44449	**KK-eiF2αi** 104562	**KK-PPP1R15** 107545	**TRiP-PPP1R15** 33011	**TRiP-4E-BPi** 80427	**UAS-4E-BP** 9147
**GMR**	N0000	N0000	N0000	E1210	E1101	E0110	E0100	N0000	N0000	N0000
**GMR PrP**	S3300	S2200	N0000	E3332	E1312	E0210	L0003	L0003	S3300	E0110

Scoring: N-No effect; S: suppressor; E: enhancer; L: lethal. Effect scored 0 (no effect) – 3 (robust change) for: size-organization-pigmentation-lethality.

Table S5. Eye phenotypes of human PrP mutants

27˚C HuPrP	**UAS-CD8-GFP**	**V129 (WT)**	**N159D (dog)**	**D167S (horse)**	**N159D D167S-2x**	**N159D D167S, N174S-3x**
**GMR**	N0000	E1200	E1200	E0100	E01000	E0100

Scoring: N-No effect; S: suppressor; E: enhancer. Effect scored 0 (no effect) – 3 (robust change) for: size-organization-pigmentation-lethality.

Disease Models & Mechanisms • Supplementary information

Table S6. Two-wayANOVA analysis for mushroom bodies

Comparisons for all pairs using Tukey-Kramer HSD

Connecting Letters Report

Level					Mean
Control-D40	A				9888.5951
Control-D1		B			6754.0952
3X-D40			C		5530.1558
V129-D1			C	D	5278.4658
2X-D1			C	D	5246.9839
N159D-D1			C	D	5102.4767
D167S-D1			C	D	5046.6443
D167S-D40			C	D	5022.8778
3X-D1			C	D	4815.1017
2X-D40				D	4566.5551
N159D-D40				E	3179.4648
V129-D40				E	2471.1883

Levels not connected by the same letter are significantly different.

Ordered Differences Report: T-test corrected

Subject 1	Subject 2	P-value	Holm p-value (0.05)	Significant (Y/N)
Control, D1	2x, D1	0.0001	0.000746269	Y
Control, D1	3x, D1	0.0001	0.000769231	Y
Control, D40	2x,d40	0.0001	0.000806452	Y
Control, D40	3x, D40	0.0001	0.000833333	Y
Control, D40	Control, D1	0.0001	0.000847458	Y
D167S, D1	Control, D1	0.0001	0.000862069	Y
D167S, d40	Control, D40	0.0001	0.000909091	Y
N159D, D1	Control, D1	0.0001	0.000925926	Y
N159D, D40	2x, D40	0.0001	0.000980392	Y
N159D, D40	3x, D40	0.0001	0.001020408	Y
N159D, D40	Control, D40	0.0001	0.00106383	Y
N159D, D40	D167S, D40	0.0001	0.001111111	Y
N159D, D40	N159D, D1	0.0001	0.001136364	Y
V129, D1	Control, D1	0.0001	0.001162791	Y

Disease Models & Mechanisms • Supplementary information

V129, D40	2x, D40	0.0001	0.001282051	Y
V129, D40	3x, D40	0.0001	0.001351351	Y
V129, D40	Control, D40	0.0001	0.001428571	Y
V129, D40	D167S, D40	0.0001	0.001515152	Y
V129, D40	V129, D1	0.0001	0.001612903	Y
3x, D40	2x, D40	0.004	0.001666667	N
3x, D40	3x, D1	0.0071	0.001785714	N
V129, D40	N159D, D40	0.0282	0.001851852	N
D167S, D40	3x, D40	0.059	0.002	N
V129, D1	3x, D1	0.0663	0.002083333	N
D167S, D40	2x, D40	0.0821	0.002173913	N
3x, D1	2x, D1	0.0867	0.002272727	N
2x, D40	2x, D1	0.087	0.002380952	N
N159D, D1	3x, D1	0.2297	0.002941176	N
V129, D1	D167S, D1	0.4034	0.004545455	N
d167s, D1	3x, D1	0.404	0.005	N
V129, D1	N159D, D1	0.4612	0.00625	N
d167s, D1	2x, D1	0.4701	0.007142857	N
N159D, D1	2x, D1	0.545	0.008333333	N
N159D, D1	D167S, D1	0.8339	0.0125	N
V129, D1	2x, D1	0.9	0.016666667	N
D167S, D40	D167S, D1	0.9328	0.025	N
